# Unraveling the early molecular and physiological mechanisms involved in response to phenanthrene exposure

**DOI:** 10.1186/s12864-016-3133-0

**Published:** 2016-10-21

**Authors:** Anne-Sophie Dumas, Ludivine Taconnat, Evangelos Barbas, Guillem Rigaill, Olivier Catrice, Delphine Bernard, Abdelilah Benamar, David Macherel, Abdelhak El Amrani, Richard Berthomé

**Affiliations:** 1 Université de Rennes 1, CNRS/OSUR/UMR 6553, Ecosystèmes-Biodiversité-Evolution, campus de Beaulieu, Bâtiment 14A, 35042 Rennes cedex, France; 2 Institute of Plant Sciences Paris Saclay (IPS2), UMR 9213/UMR1403, Université Paris Sud, CNRS, INRA, Université d’Evry, Université Paris Diderot, Sorbonne Paris Cité, Bâtiment 630, 91405 Orsay, France; 3 Laboratoire des Interactions Plantes Micro-organismes (LIPM), UMR INRA 441/CNRS 2594, CS 52627, 31326 Castanet Tolosan cedex, France; 4 Université d’Angers, UMR 1345, Institut de Recherche en Horticulture et Semences (IRHS), Bat ARES, 16 Boulevard Lavoisier, 49045 Angers cedex, France; 5 Present Address: Laboratory of Forest Genetics and Tree Breeding, AUTH, University Campus, 54124 Thessaloniki, Greece; 6 Present Address: Laboratoire de Génétique Moléculaire et de Génétique Epidémiologique, INSERM U1078, 46, rue Felix Le Dantec, CS 51819, 29218 Brest Cedex 2, France

**Keywords:** Abiotic stress, Phenanthrene, Phytoremediation, Arabidopsis, Energy availability, Transcriptome, Xenome, Metabolites

## Abstract

**Background:**

Higher plants have to cope with increasing concentrations of pollutants of both natural and anthropogenic origin. Given their capacity to concentrate and metabolize various compounds including pollutants, plants can be used to treat environmental problems - a process called phytoremediation. However, the molecular mechanisms underlying the stabilization, the extraction, the accumulation and partial or complete degradation of pollutants by plants remain poorly understood.

**Results:**

Here, we determined the molecular events involved in the early plant response to phenanthrene, used as a model of polycyclic aromatic hydrocarbons. A transcriptomic and a metabolic analysis strongly suggest that energy availability is the crucial limiting factor leading to high and rapid transcriptional reprogramming that can ultimately lead to death. We show that the accumulation of phenanthrene in leaves inhibits electron transfer and photosynthesis within a few minutes, probably disrupting energy transformation.

**Conclusion:**

This kinetic analysis improved the resolution of the transcriptome in the initial plant response to phenanthrene, identifying genes that are involved in primary processes set up to sense and detoxify this pollutant but also in molecular mechanisms used by the plant to cope with such harmful stress. The identification of first events involved in plant response to phenanthrene is a key step in the selection of candidates for further functional characterization, with the prospect of engineering efficient ecological detoxification systems for polycyclic aromatic hydrocarbons.

**Electronic supplementary material:**

The online version of this article (doi:10.1186/s12864-016-3133-0) contains supplementary material, which is available to authorized users.

## Background

Throughout the last century, industrial revolution has produced a myriad of aromatic end-products, since the increasing human activity leads to a massive use of fossil fuels, and the generation of manifold aromatic such as polycyclic aromatic hydrocarbons (PAHs). PAHs are ubiquitous products of the combustion of carbon-based substances. PAHs are one of the most widespread organic pollutants and have adverse effects on human health [1–3]. Risks associated with PAH pollution can be partially overcome by removing them from the soil using chemical, physical or thermal treatments. These techniques affect the environment and often relocate the pollutant from one compartment to another [4]. As an example, in physico-chemical remediation technologies, PAHs in contaminated soils are removed using a mixture of water and co-solvents. PAHs are transfered in the fluid and a secondary treatment of the extract is necessary.

Alternative removal solutions lie in “green technologies” which make use of the natural ability of living organisms to transform pollutants into less harmful compounds [5]. The development of these approaches, including bioremediation and phytoremediation, has also stimulated studies dedicated to identifying factors behind efficient PAH remediation. PAH susceptibility to biological detoxification is correlated with (i) their adsorption on organic matter that determines their availability and (ii) their structure, composed of two or more benzene rings, with “light” PAHs (2–3 rings) being more efficiently degraded than more complex “heavy” PAHs [6, 7]. Furthermore, PAH detoxification varies with environmental conditions, plant species or the nature of plant-microbe interactions in the rhizosphere [8–12]. Improvement in PAH bioremediation technologies can draw on studies of plant-microbe interactions in the soil and genetic engineering of plants able to stimulate these interactions and/or directly degrade PAHs [13–16].

Development of such innovative tools for phytoremediation of PAHs remains scarce mainly because cellular and molecular mechanisms involved in uptake and metabolization remain poorly understood [12], in contrast with the numerous studies on bioremediation agents (bacteria, fungi and algae); [17–22]. However, molecular processes involved in phytoremediation are based on similarities with the xenobiotic detoxification systems described in the mammalian liver [23, 24]. Thus, Edwards et al. [24] defined the xenome as “the biosystem responsible for the detection, transport and detoxification of xenobiotics in the plant cell”.

Some PAHs can be metabolized in cell cultures of different plant species and appear to be conjugated to soluble sugars or linked to glutathione [25, 26]. Phenanthrene (PHN), is taken up by roots in Arabidopsis [27] and in wheat [28, 29] implying that this pollutant or its derivatives can be transported through the plant. Interestingly, wheat roots uptake PHN by rapid passive diffusion through aquaglyceroporins in cell membranes just after the transfer of the plant to PHN-supplemented medium, and by slow active absorption, probably via a PHN/H+ symporter, after 2 h of incubation [29]. These observations suggest that PHN absorption and its putative transformation can be quickly regulated by the plant. However actors involved in such early plant response to PHN are still unknown. Indeed, most of these studies have been carried out using long-term (14–30 days) PHN exposures [27, 30, 31]. Long-term PHN exposure alters organelle structure, plant morphology and induces the expression of genes encoding proteins with antioxidant activities [27, 31]. Some features are shared between plant responses to PAHs, other abiotic stresses and pathogens. In particular, the production of reactive oxygen species (ROS) appears to play an important role in stress-related phenotypes observed following PHN treatments [31] and in the transcriptional response to long-term exposure to PHN [30]. Putative oxidation of PHN by mono- or di-oxygenases, which remain to be identified, may trigger an increase in ROS levels and induce expression of protein-coding genes implicated in the control of oxidative stress [30].

The purpose of this study was to screen the molecular events involved in the early plant response to PHN. Kinetic analysis of the transcriptome led to the identification of differentially expressed genes that may be involved in PHN detoxification. Through physiological characterization and titration of metabolites, we show that PHN accumulation inhibits electron transfer and photosynthesis within a few minutes, strongly suggesting that energy transformation is the crucial limiting factor that leads the plant to exhaustion after PHN exposure.

## Results

### PHN exposure affects plant development in a dose-dependent manner

**Fig. 1:**
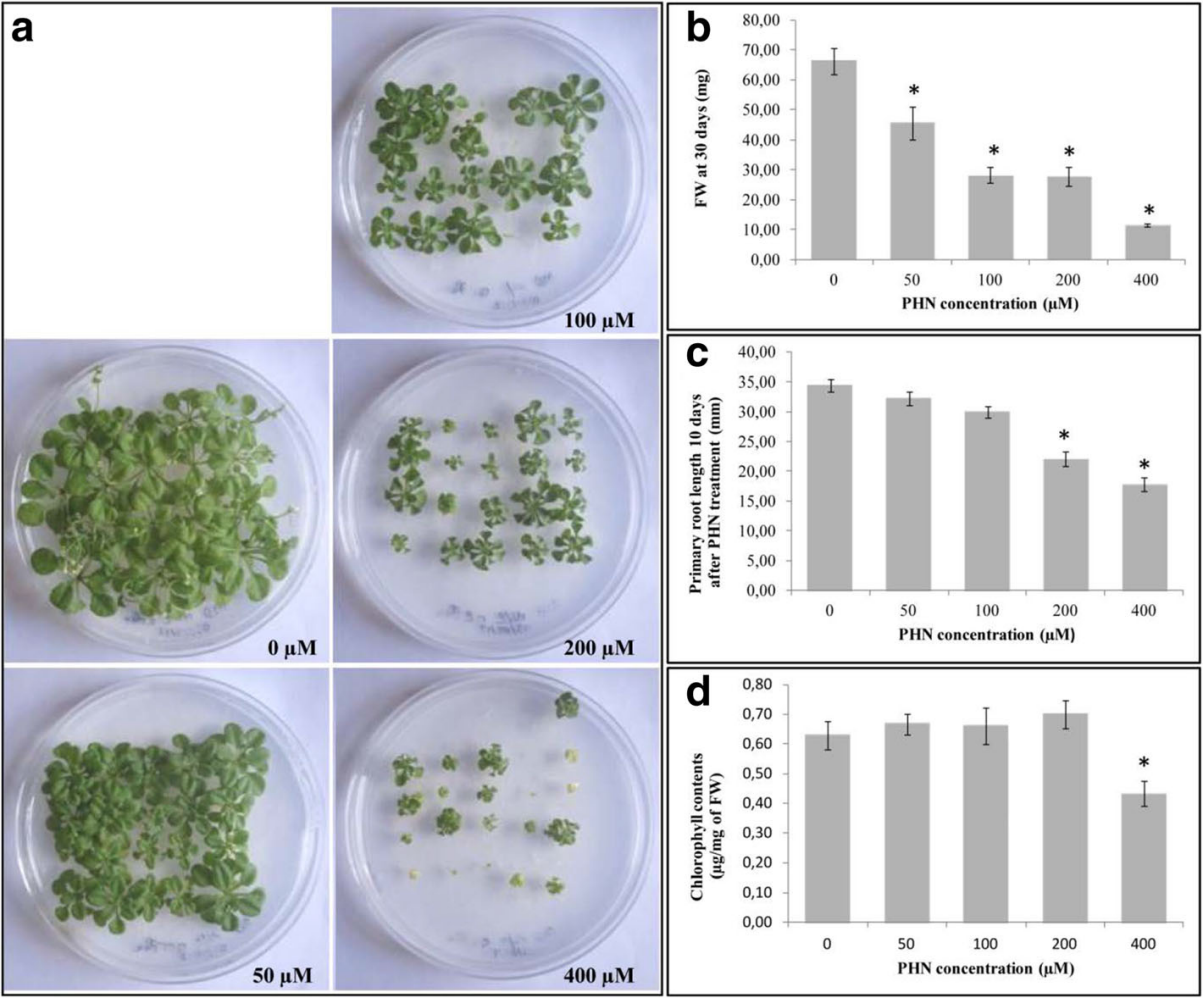
Impact of PHN on plant development. *A. thaliana* plantlets were grown on MS/2 sucrose free medium supplied with 0- (DMSO control), 50-, 100-, 200- or 400 μM of PHN. 30-days old plantlets were phenotyped (**a**) and harvested to measure their fresh weight (**b**) and chlorophyll contents (**d**). Values correspond to the means of four biological replicates for which three plant shoots were used. Standard errors of the means (SEM) are indicated. Plantlets were also grown vertically and primary root length measured after 10 days of growth (**c**). The indicated values correspond to the means of at least 18 independent measurements and bars represent SEM. *indicates a significant difference between treated and control plants (i.e. *p*-value < 0.05)

Experiments were performed using sucrose-free medium. In PHN treatments shorter than 24 h, no macroscopic alterations were observed. However, compared to the control, 30 days PHN exposure inhibited plant shoot development, and heterogeneous phenotypes were observed within the same petri dish, even at the lowest concentration (Fig. 1a). The dose–response phenotype was always correlated with a significant decrease in shoot fresh weight (Fig. 1b) and was characterized by a significant reduction in primary root length for 200 and 400 μM PHN treatments (Fig. 1c). The strongest effect was observed at 400 μM, with an increase in the number of chlorotic plants that failed to develop. Quantification of the chlorophyll content confirmed a significant decrease in chlorophyll at high PHN concentrations (Fig. 1d). We therefore decided to study early response to PHN using the sub-lethal concentration of 200 μM, above which chlorophyll content of plant leaves significantly decreases after long-term exposure. To avoid any heterogeneous phenotype that may be correlated with the low aqueous solubility of PHN and its gradient concentration effects in solid medium, plantlets were incubated in liquid medium, with PHN or DMSO for the transcriptome analysis.

### Accumulation of PHN in planta and histolocalization

**Fig. 2:**
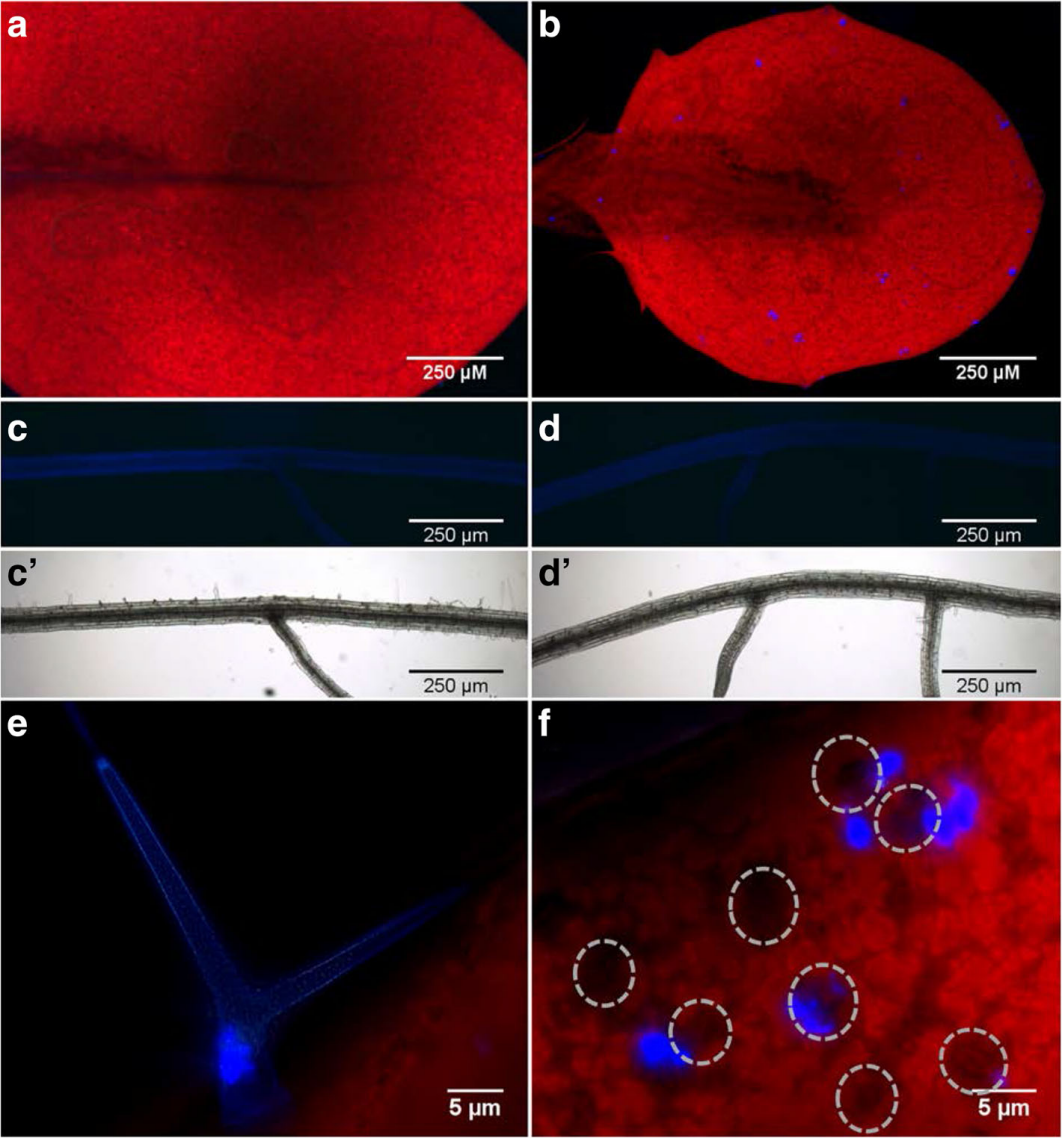
Detection of PHN using fluorescence microscopy. Plants were grown for 15 days on MS/2 sucrose-free medium and then transferred for 5 days on an identical medium supplemented with DMSO as a control (**a**, **c**, **c’**) or 200 μM PHN (**b**, **d**, **d’**, **e**, **f**). **a**-**f** panels show representative observations of samples under UV light. **c’**, **d’** panels show bright field observations corresponding to the primary roots of C and D panels. Third leaf of control (**a**) and PHN-treated plants (**b**). Primary root of control (**c**) and PHN-treated plants (**d**). Bright spots corresponding to PHN observed in trichomes on the adaxial side of leaves (**e**). PHN aggregates on the epidermis on the abaxial side of leaves near stomatal guard cells, indicated by Circles (**f**)

The ability of Arabidopsis to absorb PHN from a solid medium was firstly tested by gas chromatography and mass spectrometry analysis. Accumulation of PHN in plant ranged from at 200 to 300 μg/g of dry plant tissue after 24 h of exposure. In parallel, we investigated putative PHN transport and localization in tissues and cells using 15-day-old plantlets grown vertically in vitro and transferred for five days on solid medium with only roots being in contact with the medium. To setup fluorescence detection assay, spectral properties and emission lines of PHN were determined using PHN solubilized in various DSMO solutions (Additional file 1: Figures S1 and S2). We used the PHN-specific 430 nm emission line to detect it in subsequent experiments. Although fluorescence was not detected in leaves and roots of control plants (Fig. 2a and c), PHN fluorescence, confirmed by spectra, was only detected on the epidermis and in the trichomes of leaves (Fig. 2b, e and f) of PHN-treated plants. Bright spots corresponding to PHN were only found in trichomes on the adaxial side of leaves (Fig. 2e), whereas PHN aggregates were always observed on the epidermis surface (Additional file 1: Figure S3), in the vicinity of stomatal guard cells on the abaxial side of leaves (Fig. 2f).

### Short PHN exposure induces a rapid transcriptional reprogramming

To determine the early molecular events involved in the plant response to PHN exposure, the experimental design was set up to compare 0 min with 30 min, 30 min with 2 h, 2 h with 4 h, 4 h with 8 h and 8 h with 24 h of incubation with PHN or DMSO and to compare PHN treatment with the control at each time point (Additional file 1: Figure S4A). Overall, 1262 genes were differentially expressed in PHN-treated plants, with 58, 130, 174, 650 and 897 genes being differentially expressed at 30 min, 2, 4, 8 and 24 h, respectively. An ANOVA analysis helped to select significantly differentially expressed genes (DEGs) (Additional file 2: Table S3) showing an interaction between time and treatment. 467 genes were recovered, with 14, 47, 77, 275 and 360 genes differentially expressed after 30 min, 2, 4, 8 and 24 h of PHN treatment, respectively. Using real-time RT-PCR, we confirmed expression changes for 11 genes analyzed (Additional file 1: Figure S5).

### Rapid PHN transcriptional reprogramming identified two distinct main steps

**Fig. 3:**
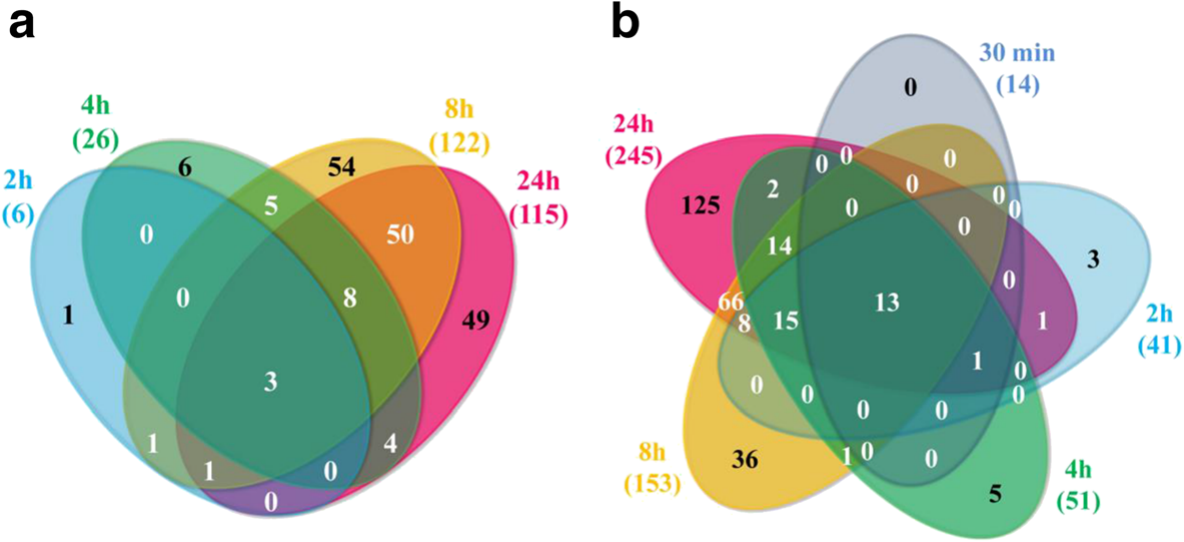
Venn diagrams of the total number of differentially expressed genes (DEGs), selected by ANOVA analysis (*p*-value < 0.05), showing an interaction between time and treatment. The indicated sets of genes correspond to downregulated (**a**) and upregulated genes (**b**) obtained by comparing phenanthrene (PHN)-treated samples with control samples at 30 min, 2, 4, 8 and 24 h. DEGs listed in Additional file 2: Table S3 were selected by statistical analysis using the Bonferroni method with a *p*-value cut-off of 0.05. Raw transcriptomic data are available in Additional file 2: Table S1

All 14 DEGs at 30 min were upregulated. For the other comparisons, upregulated genes accounted for the majority of the genes whose expression was modified (41/47, 51/77, 153/275, 245/360 at 2, 4, 8, 24 h, respectively). Venn diagrams were generated with the DEG lists for each time point (Fig. 3a and b). Few genes were specifically differentially regulated at 30 min, 2 and 4 h (0/3/5 up- and 0/1/6 downregulated genes at 30 min, 2 and 4 h, respectively), contrasting with comparisons at 8 and 24 h (36/125 up- and 44/59 downregulated genes at 8 and 24 h respectively). Strikingly, most of the genes upregulated at 30 min were also upregulated at later time points, with increasing accumulation of the corresponding transcript over time. The high number of DEGs in common between the 8 h and 24 h time points (50 downregulated and 66 upregulated) and the high significance of this overlap (*p*-value < 0.0001) suggest that the same pathways were regulated in response to PHN after 4 h of treatment. The strong increase in the number of DEGs after 4 h of PHN exposure and the weak overlap between DEGs identified after 30 min, 2 and 4 h compared with 8 h and 24 h of treatment reflect that Arabidopsis responses to PHN are subdivided into an early rapid response from 30 min to 4 h followed by substantial transcriptional reprogramming from 8 to 24 h (Fig. 3a and b). Only five genes encoding a plant thionin, a putative aspartyl protease, the cytochrome P450 protein CYP704B1, the glycosyl hydrolase ATXYN1 and the senescence protein ATWI-12, displayed opposite regulation patterns between 4 and 24 h of treatment (*AT1G58370*, *AT1G66100*, *AT1G69500*, *AT3G10985* and *AT5G48430* respectively). Table 1 shows the 20 most kinetically regulated DEGs, when available.

**Table 1:** The most differentially expressed genes (DEGs) during the time course in PHN-treated plants

AGI identifier	Gene annotation	30 min		2 h		4 h		8 h		24 h	
		Ratio	*p*-value	Ratio	*p*-value	Ratio	*p*-value	Ratio	*p*-value	Ratio	*p*-value
***Genes rapidly upregulated after 30 min of treatmentwhose levles remained high throughout the time course***											
AT1G70800	EHB1__Calcium-dependent lipid-binding (CaLB domain) family protein	**0.64**	***4*** **.** ***88E-02***	**1.23**	***2.80E-09***	**1.91**	***0.00E + 00***	**1.97**	***0.00E + 00***	**1.19**	***0.00E + 00***
AT4G38620^a^	ATMYB4_MYB4_myb domain protein 4	**3.51**	***0.00E + 00***	**2.19**	***0. 00E + 00***	**2.30**	***0.00E + 00***	**2.18**	***0.00E + 00***	**1.94**	***0.00E + 00***
AT2G47950	unknown protein	**1.49**	***0.00E + 00***	**2.27**	***0.00E + 00***	**2.27**	***0.00E + 00***	**2.65**	***0.00E + 00***	**1.91**	***0.00E + 00***
AT5G48540^a^	receptor-like protein kinase-related family protein	**1.42**	***0.00E + 00***	**2.44**	***0.00E + 00***	**3.27**	***0.00E + 00***	**3.48**	***0.00E + 00***	**3.51**	***0.00E + 00***
AT5G59820^a^	RHL41_ZAT12__C2H2-type zinc finger family protein	**1.19**	***0.00E + 00***	**1.63**	***0.00E + 00***	**2.14**	***0.00E + 00***	**2.70**	***0.00E + 00***	**2.50**	***0.00E + 00***
AT2G16900	Arabidopsis p hosp holip ase-like protein (PEARLI 4) family	**0.98**	***3.61E-09***	**1.68**	***0.00E + 00***	**1.67**	***3.06E-11***	**1.61**	***0.00E + 00***	**2.16**	***0.00E + 00***
**AT1G68620** ^**b**^	**alpha/beta-Hydrolases superfamily protein**	**0.93**	***6. 02E-08***	**2.18**	***0.00E + 00***	**1.68**	***2.29E-11***	**2.77**	***0.00E + 00***	**2.70**	***0.00E + 00***
AT5G56630^b^	PFK7__phosphofructokinase 7	**0.91**	***2.45E-07***	**1.59**	***0.00E + 00***	**2.41**	***0.00E + 00***	**1.54**	***0.00E + 00***	**2.53**	***0.00E + 00***
AT3G22840^a^	ELIP_ELIP1__Chlorophyll A-B binding family protein	**0.89**	***6.36E-07***	**1.40**	***0.00E + 00***	**2.19**	***0.00E + 00***	**1.70**	***0.00E + 00***	**2.38**	***0.00E + 00***
AT1G63840	RING/U-box superfamily protein	**0.83**	***1.31E-05***	**1.80**	***0.00E + 00***	**1.64**	***1.22E-10***	**2.10**	***0.00E + 00***	**2.16**	***0.00E + 00***
AT2G36590	ATPROT3_ProT3__proline transporter 3	**0.79**	***7. 54E-05***	**1.42**	***0.00E + 00***	**1.93**	***0.00E + 00***	**1.80**	***0.00E + 00***	**2.24**	***0.00E + 00***
AT4G39670	Glycolipid transfer protein (GLTP) family protein	**0.73**	***1.16E-03***	**1.26**	***9.30E-10***	**1.01**	***4.52E-02***	**1.42**	***0.00E + 00***	**2.13**	***0.00E + 00***
AT4G27657	unknown protein	**0.70**	***4.31E-03***	**1.26**	***6.61E-10***	**1.10**	***4.30E-03***	**1.75**	***0.00E + 00***	**0.74**	***2.19E-03***
AT4G15248^a^	B-box type zinc fnger family protein	**1.39**	***0.00E + 00***	**1.75**	***0.00E + 00***	**1.04**	***1.93E-02***	**0**.35	*1.00E + 00*	**1.63**	***0.00E + 00***
***Genes differentially upregulated after 2 h whose levels remained high***											
AT2G35980	ATNHL10_NHL10_YLS9__Late embryogenesis abundant (LEA) hydroxyproline-rich glycoprotein family	0.56	*7.20E-01*	**1.72**	**0.00E + 00**	**1.96**	***0.00E + 00***	**1.81**	***0.00E + 00***	**2.34**	***0.00E + 00***
AT3G22600	Bifunctional inhibitor/lipid-transfer protein/seed storage 2S albumin superfamily protein	0.16	*1.00E + 00*	**1.68**	***0.00E + 00***	**1.96**	***0.00E + 00***	**1.36**	***0.00E + 00***	**1.96**	***0.00E + 00***
AT4G25640	ATDTX35_DTX35_FFT__detoxifying efflux carrier 35	0.27	*1.00E + 00*	**1.66**	***0.00E + 00***	**1.40**	***6.73E-07***	**1.98**	***0.00E + 00***	**2.11**	***0.00E + 00***
**AT3G21560**	**UGT84A2__UDP-Glycosyltransferase superfamily protein**	0.52	*1.00E + 00*	**1.48**	***0.00E + 00***	**2.21**	***0.00E + 00***	**1.60**	***0.00E + 00***	**1.93**	***0.00E + 00***
AT1G74010^b^	Calcium-dependent phosphotriesterase superfamily protein	0.46	*1.00E + 00*	**1.39**	***0.00E + 00***	**1.68**	***3.06E-11***	**1.32**	***0.00E + 00***	**2.50**	***0.00E + 00***
AT1G75040	PR-5_PR5__pathogenesis-related gene 5	0.21	*1.00E + 00*	**1.32**	***3.84E-11***	**1.50**	***2.24E-08***	**1.71**	***0.00E + 00***	**1.75**	***0.00E + 00***
AT1G30700	FAD-binding Berberine family protein	0.48	*1.00E + 00*	**1.26**	***9. 30E-10***	**1.06**	***1.24E-02***	**1.56**	***0.00E + 00***	**2.28**	***0.00E + 00***
AT3G04300	RmlC-like cupins superfamily protein	0.37	*1.00E + 00*	**1.25**	***1.14E-09***	**1.73**	***0.00E + 00***	**2.08**	***0.00E + 00***	**2.25**	***0.00E + 00***
AT1G18980	RmlC-like cupins superfamily protein	0.2	*1.00E + 00*	**1.16**	***6.26E-08***	**1.55**	***3.16E-09***	**1.57**	***0.00E + 00***	**1.59**	***0.00E + 00***
AT1G76980	unknown protein	0.51	*1.00E + 00*	**1.15**	***9.70E-08***	**1.67**	***4.59E-11***	**1.78**	***0.00E + 00***	**1.85**	***0.00E + 00***
AT5G54500^b^	FQR1__flavodoxin-like quinone reductase 1	0.47	*1.00E + 00*	**1.12**	***3.41E-07***	**1.14**	***1.72E-03***	**1.50**	***0.00E + 00***	**1.79**	***0.00E + 00***
AT1G74450	Protein of unknown function (DUF793)	0.41	*1.00E + 00*	**1.09**	***1.17E-06***	**1.16**	***9.83E-04***	**1.06**	***3.11E-09***	**1.10**	***2.24E-11***
AT3G04000^b^	NAD(P)-binding Rossmann-fold superfamily protein	0.06	*1.00E + 00*	**0.95**	***3.38E-04***	**1.16**	***1.01E-03***	**1.53**	***0.00E + 00***	**1.23**	***0.00E + 00***
AT1G27120	Galactosyltransferase family protein	0.00	*1.00E + 00*	**0.91**	***1.33E-03***	**2.00**	***0.00E + 00***	**2.30**	***0.00E + 00***	**2.45**	***0.00E + 00***
AT2G17500	Auxin efflux carrier family protein	−0.20	*1.00E + 00*	**0.80**	***4.29E-02***	**1.19**	***3.99E-04***	**1.79**	***0.00E + 00***	**1.20**	***0.00E + 00***
***Genes differentially upregulated after 4 h of treatment***											
**AT1G78340**	**ATGSTU22_GSTU22__glutathione S-transferase TAU 22**	0.04	*1.00E + 00*	0.56	*1.00E + 00*	**1.62**	***2.45E-10***	**1.75**	***0.00E + 00***	**1.69**	***0.00E + 00***
***AT1G17170***	**ATGSTU24_GST_GSTU24__glutathione S-transferase TAU 24**	0.06	*1. 00E + 00*	0.39	*1. 00E + 00*	**1.57**	***1.98E-09***	**2.00**	***0.00E + 00***	**2.05**	***0.00E + 00***
AT3G51660	Tautomerase/MIF superfamily protein	0.10	*1. 00E + 00*	0.75	*1.85E-01*	**1.47**	***5.93E-08***	**1.8**	***0.00E + 00***	**2.27**	***0.00E + 00***
**AT5G36270** ^**b**^	**similar to DHAR2, glutathione dehydrogenase (ascorbate)**	0.00	*1. 00E + 00*	0.55	*1.00E + 00*	**1.38**	***1.16E-06***	**1.56**	***0.00E + 00***	**1.74**	***0.00E + 00***
**AT4G15480**	**UGT84A1__UDP-Glycosyltransferase superfamily protein**	0.18	*1.00E + 00*	0.47	*1.00E + 00*	**1.27**	***4.29E-05***	**1.02**	***2.61E-08***	**1.40**	***0.00E + 00***
**AT1G78380**	**ATGSTU19_GST8_GSTU19__glutathione S-transferase TAU 19**	0.1	*1. 00E + 00*	0.69	*1.00E + 00*	**1.23**	***1.42E-04***	**1.45**	***0.00E + 00***	**1.49**	***0.00E + 00***
***AT2G29500***	HSP20-like chaperones superfamily protein	0.16	*1. 00E + 00*	0.29	*1.00E + 00*	**1.15**	***1.22E-03***	**1.11**	***1.94E-10***	**1.53**	***0.00E + 00***
***AT1G75030***	ATLP-3_TLP-3__thaumatin-like protein 3	0.19	*1.00E + 00*	0.59	*1.00E + 00*	**1.13**	***1.98E-03***	**1.45**	***0.00E + 00***	**1.35**	***0.00E + 00***
**AT1G75270** ^**b**^	**DHAR2__dehydroascorbate reductase 2**	0.36	*1.00E + 00*	0.78	*8.04E-02*	**1.09**	***5.83E-03***	**1.60**	***0.00E + 00***	**1.49**	***0.00E + 00***
**AT1G64900**	**CYP89_CYP89A2__cytochrome P450, family 89, subfamly A, polypeptide 2**	−0.03	*1. 00E + 00*	0.33	*1.00E + 00*	**1.08**	***6.73E-03***	**1.16**	***1.49E-11***	**1.19**	***0.00E + 00***
AT3G13520^b^	AGP12_ATAGP12__arabinogalactan protein 12	0.17	*1. 00E + 00*	0.57	*1.00E + 00*	**1.08**	***6.96E-03***	**1.39**	***0.00E + 00***	**0.88**	***4.96E-06***
**AT2G12190**	**Cytochrome P450 superfamily protein**	−0.04	*1.00E + 00*	0.43	*1.00E + 00*	**1.04**	***1.98E-02***	**0.97**	***3.77E-07***	**0.96**	***6.31E-08***
AT4G13180^b^	NAD(P)-binding Rossmann-fold superfamily protein	0.28	*1.00E + 00*	0.29	*1.00E + 00*	**1.03**	***2.87E-02***	**1.07**	***1.53E-09***	**1.39**	***0.00E + 00***
**AT1G05680**	**UGT74E2__Uridine diphosphate glycosyltransferase 74E2**	0.14	*1.00E + 00*	−0.05	*1.00E + 00*	**1.01**	***4.09E-02***	**1.8**	***0.00E + 00***	**0.83**	***3.77E-05***
AT2G48140	EDA4 Bifunctional inhibitor/lipid-transfer protein/seed storage 2S albumin superfamily protein	0.02	*1. 00E + 00*	0.74	*2.24E-01*	**1.20**	***2.82E-04***	**0.73**	***9.70E-03***	0.40	*1.00E + 00*
**AT3G09270**	**ATGSTU8_GSTU8__glutathione S-transferase TAU 8**	−0.12	*1. 00E + 00*	0.41	*1.00E + 00*	**1.15**	***1.12E-03***	0.26	*1.00E + 00*	**1.09**	***5.98E-11***
AT1G23490	ARF 1_ATARF_ATARF 1_ATARFA1A__ADP-ribosylation fictor 1	0.48	*1.00E + 00*	0.76	*1 49E-01*	**1.04**	***1.99E-02***	0.64	*2.29E-01*	**1.32**	***0.00E + 00***
**ATI G58370** ^**b**^	**ATXYN1_RXF12_glycosyl hydrolase famly 10 protein/carbohydrate-binding domain-containing**	0.17	*1.00E + 00*	0.64	*1.00E + 00*	**1.13**	***2.13E-03***	0.08	*1.00E + 00*	**−0.86**	***1.10E-05***
***Genes differentially upregulated after 8 h of treatment***											
AT5G22140	FAD/NAD(P)-binding oxidoreductase family protein	0.3	*1. 00E + 00*	0.02	*1.00E + 00*	0.76	*1.00E + 00*	**2.15**	***0.00E + 00***	**1.97**	***0.00E + 00***
AT3G44190	FAD/NAD(P)-binding oxidoreductase family protein	0.03	*1. 00E + 00*	0.21	*1.00E + 00*	0.95	*1.78E-01*	**2.05**	***0.00E + 00***	**2.01**	***0.00E + 00***
AT2G23110	Late embryogenesis abundant protein, group 6	0.45	*1.00E + 00*	0.78	*8.62E-02*	0.51	*1.00E + 00*	**1.87**	***0.00E + 00***	**2.04**	***0.00E + 00***
AT5G64250	Aldolase-type TIM barrel family protein	−0.01	*1.00E + 00*	0.35	*1.00E + 00*	0.59	*1.00E + 00*	**1.62**	***0.00E + 00***	**1.55**	***0.00E + 00***
AT3G28210^a^	PMZ_SAP12__zinc finger (AN1-like) family protein	0.36	*1. 00E + 00*	0.52	*1.00E + 00*	0.98	*8.17E-02*	**1.60**	***0.00E + 00***	**2.09**	***0.00E + 00***
AT1G75280^b^	NmrA-like negative transcriptional regulator family protein	0.15	*1. 00E + 00*	0.62	*1.00E + 00*	0.86	*1.00E + 00*	**1.56**	***0.00E + 00***	**1.37**	***0.00E + 00***
**AT4G24160**	**alpha/beta-Hydrolases superfamily protein**	−0.01	*1.00E + 00*	0.40	*1.00E + 00*	0.59	*1.00E + 00*	**1.52**	***0.00E + 00***	**1.25**	***0.00E + 00***
AT3G10500	anac053_NAC053__NAC domain containing protein 53	0.12	*1.00E + 00*	0.77	*1.19E-01*	0.66	*1.00E + 00*	**1.48**	***0.00E + 00***	**1.16**	***0.00E + 00***
AT2G01180^a^	ATLPP1_ATPAP1_LPP1_PAP1__phosphatidic acid phosphatase 1	0.20	*1. 00E + 00*	0.33	*1.00E + 00*	0.95	*1.92E-01*	**1.44**	***0.00E + 00***	**1.97**	***0.00E + 00***
AT5G54206	similar to 12-oxophytodienoate reductase OPR1	0.17	*1. 00E + 00*	0.15	*1.00E + 00*	0.29	*1.00E + 00*	**1.41**	***0.00E + 00***	**2.04**	***0.00E + 00***
**AT2G29460** ^**b**^	**ATGSTU4_GST22_GSTU4__glutathione S-transferase tau 4**	0.12	*1.00E + 00*	0.52	*1.00E + 00*	1.00	*5.79E-02*	**1.37**	***0.00E + 00***	**0.95**	***1.12E-07***
**AT4G34131**	**UGT73B3__UDP-glucosyl transferase 73B3**	0.33	*1.00E + 00*	0.38	*1.00E + 00*	0.89	*7.10E-01*	**1.31**	***0.00E + 00***	**1.38**	***0.00E + 00***
AT1G05670	Pentatricopeptide repeat (PPR-like) superfamily protein	0.20	*1. 00E + 00*	0.02	*1.00E + 00*	0.71	*1.00E + 00*	**1.30**	***0.00E + 00***	**0.69**	***1.70E-02***
AT1G77120^b^	ADH_ADH1_ATADH_ATADH1__alcohol dehydrogenase 1	−0.18	*1. 00E + 00*	0.54	*1.00E + 00*	0.25	*1.00E + 00*	**1.30**	***0.00E + 00***	**1.02**	***3.44E-09***
AT2G21620	RD2__Adenine nucleotide alpha hydrolases-like superfamily protein	−0.01	*1.00E + 00*	0.75	*1.73E-01*	0.94	*2.14E-01*	**1.28**	***0.00E + 00***	**1.82**	***0.00E + 00***
AT1G66580	RPL10C_SAG24__senescence associated gene 24	0.32	*1.00E + 00*	0.45	*1.00E + 00*	0.63	*1.00E + 00*	**1.26**	***0.00E + 00***	**1.44**	***0.00E + 00***
AT2G36950	Heavy metal transport/detoxification superfamily protein	0.21	*1. 00E + 00*	0.45	*1.00E + 00*	−0.10	*1.00E + 00*	**1.26**	***0.00E + 00***	**1.01**	***4.60E-09***
AT1G72490	unknown protein	−0.09	*1. 00E + 00*	0.20	*1.00E + 00*	0.02	*1.00E + 00*	**1.25**	***0.00E + 00***	**1.10**	***2.24E-11***
AT1G67600^a^	Acid phosphatase/vanadium-dependent haloperoxidase-related protein	0.01	*1.00E + 00*	0.45	*1.00E + 00*	0.5	*1.00E + 00*	**1.25**	***0.00E + 00***	**1.07**	***1.94E-10***
AT5G27760	Hypoxia-responsive family protein	0.14	*1.00E + 00*	0.47	*1.00E + 00*	0.83	*1.00E + 00*	**1.19**	***0.00E + 00***	**1.43**	***0.00E + 00***
***Genes differentially upregulated after 24 h of treatment***											
AT1G65290b	mtACP2__mitochondrial acyl carrier protein 2	0.13	*1.00E + 00*	0.06	*1.00E + 00*	0.13	*1.00E + 00*	0.34	*1.00E + 00*	**2.63**	***0.00E + 00***
AT4G3 7990^b^	ATCAD8_CAD-B2_ELI3_ELI3-2__elicitor-activated gene 3-2	0.07	*1.00E + 00*	0.3	*1.00E + 00*	0.34	*1.00E + 00*	0.00	*1.00E + 00*	**2.43**	***0.00E + 00***
AT5G25260	SPFH/Band 7/PHB domain-containing membrane-associated protein family	−0.1	*1.00E + 00*	−0.06	*1.00E + 00*	0.30	*1.00E + 00*	0.04	*1.00E + 00*	**2.07**	***0. 00E + 00***
AT2G18690	unknown protein	0.11	*1.00E + 00*	0.70	*7.28E-01*	0.54	*1.00E + 00*	0.66	*1.32E-01*	**1.98**	***0. 00E + 00***
AT4G12490	Bifunctional inhibitor/lipid-transfer protein/seed storage 2S albumin superfamily protein	0.22	*1.00E + 00*	0.07	*1.00E + 00*	0.66	*1.00E + 00*	0.28	*1.00E + 00*	**1.90**	***0.00E + 00***
AT1G14870	AtPCR2_PCR2_PLANT CADMIUM RESISTANCE 2	−0.03	*1.00E + 00*	0.51	*1.00E + 00*	0.03	*1.00E + 00*	0.48	*1.00E + 00*	**1.76**	***0 00E + 00***
AT2G23150	ATNRAMP3_NRAMP3__natural resistance-associated macrophage protein 3	−0.17	*1.00E + 00*	−0.16	*1.00E + 00*	0.01	*1.00E + 00*	0.59	*1.00E + 00*	**1.69**	***0 00E + 00***
AT2G17740	Cysteine/Histidine-rich C1 domain family protein	−0.03	*1.00E + 00*	−0.15	*1.00E + 00*	0.66	*1.00E + 00*	0.20	*1.00E + 00*	**1.67**	***0 00E + 00***
AT1G66090	Disease resistance protein (TIR-NBS class)	0.36	*1.00E + 00*	0.29	*1.00E + 00*	0.66	*1.00E + 00*	0.55	*1.00E + 00*	**1.67**	***0 00E + 00***
AT5G06320	NHL3_NDR1/HN1-Ike 3	−0.28	*1.00E + 00*	0.11	*1.00E + 00*	−0.08	*1.00E + 00*	0.31	*1.00E + 00*	**1.60**	***0 00E + 00***
AT2G29350	SAG13__senescence-associated gene 13	−0.17	*1.00E + 00*	0.17	*1.00E + 00*	0.82	*1.00E + 00*	0.59	*1.00E + 00*	**1.55**	***0 00E + 00***
AT1G13330	AHP2__Arabidopsis Hop2 homolog	0.00	*1.00E + 00*	−0.09	*1.00E + 00*	0.69	*1.00E + 00*	0.36	*1.00E + 00*	**1.55**	***0 00E + 00***
AT5G13320	GDG1_GH3.12_PBS3_WIN3__Auxin-responsive GH3 family protein	−0.1	*1.00E + 00*	−0.25	*1.00E + 00*	0.11	*1.00E + 00*	−0.07	*1.00E + 00*	**1.54**	***0 00E + 00***
AT5G17380^b^	Thiamine pyrophosphate dependent pyruvate decarboxylase family protein	0.04	*1.00E + 00*	0.03	*1.00E + 00*	0.35	*1.00E + 00*	0.65	*1.76E-01*	**1.48**	***0 00E + 00***
AT2G20142^a^	Toll-Interleukin-Resistance (TIR) domain family protein	0.06	*1.00E + 00*	0.05	*1.00E + 00*	0.21	*1.00E + 00*	0.23	*1.00E + 00*	**1.47**	***0 00E + 00***
AT4G26200	ACS7_ATACS7__1-amino-cyclopropane-1-carboxylate synthase 7	0.22	*1.00E + 00*	−0.01	*1.00E + 00*	0.18	*1.00E + 00*	0.26	*1.00E + 00*	**1.44**	***0 00E + 00***
AT1G74710	ATICS1_EDS16_ICS1_SID2__ADC synthase superfamily protein	0.05	*1.00E + 00*	−0.19	*1.00E + 00*	0.14	*1.00E + 00*	−0.09	*1.00E + 00*	**1.41**	***0 00E + 00***
AT4G11890	Protein kinase superfamily protein	0.10	*1.00E + 00*	0.19	*1.00E + 00*	−0.04	*1.00E + 00*	0.56	*1.00E + 00*	**1.38**	***0 00E + 00***
AT5G25250	SPFH/Band 7/PHB domain-containing membrane-associated protein family	0.07	*1.00E + 00*	0.00	*1.00E + 00*	0.28	*1.00E + 00*	0.40	*1.00E + 00*	**1.38**	***0.00E + 00***
AT1G26380	FAD-binding Berberine family protein	0.24	*1.00E + 00*	0.24	*1.00E + 00*	0.34	*1.00E + 00*	0.43	*1.00E + 00*	**1.38**	***0 00E + 00***
***Genes differentially down-regulated after 2 h of treatment whose levels remained low***											
AT1G26810	GALT1__galactosyltransferase1	−0.13	*1.00E + 00*	**−1.16**	***7.36E-08***	**−1.71**	***7.64E-12***	**−1.65**	***0.00E + 00***	**−1.79**	***0 00E + 00***
AT3G19450^b^	ATCAD4_CAD_CAD-C_CAD4__GroES-like zinc-binding alcohol dehydrogenase family protein	0.03	*1.00E + 00*	**−0.82**	***2.57E-02***	**−1.22**	***1.62E-04***	**−1.74**	***0.00E + 00***	**−1.74**	***0.00E + 00***
AT5G48930^b^	HCT__hydroxycinnamoyl-CoA shikimate/quinate hydroxycinnamoyl transferase	−0.20	*1.00E + 00*	**−1.17**	***5.58E-08***	**−1.11**	***3.21E-03***	**−1.50**	***0.00E + 00***	**−1.18**	***0 00E + 00***
***Genes differentially downregulated after 4 h of treatment whose levels remained low***											
AT1G08630b	THA1__threonine aldolase 1	0.06	*1.00E + 00*	−0.54	*1.00E + 00*	***−1.81***	***0.00E + 00***	***−0.92***	***3.97E-06***	**−1.62**	***0 00E + 00***
AT1G43160^a^	RAP2.6__related to AP2 6	0.02	*1.00E + 00*	−0.57	*1.00E + 00*	***−1.57***	***1.81E-09***	***−1.16***	***7.45E-12***	**−0.99**	***1.74E-08***
AT5G49730	ATFRO6_FRO6_FRO6__ferric reduction oxidase 6	−0.05	*1.00E + 00*	−0.27	*1.00E + 00*	***−1.35***	***3.59E-06***	***−0.79***	***9.47E-04***	**−1.46**	***0 00E + 00***
AT5G25460	Protein of unknown function, DUF642	−0.03	*1.00E + 00*	−0.55	*1.00E + 00*	***−1.30***	***1.38E-05***	***−1.72***	***0.00E + 00***	**−1.41**	***0 00E + 00***
AT4G29905	unknown protein	0.00	*1.00E + 00*	0.29	*1.00E + 00*	***−1.16***	***9.80E-04***	***−0.76***	***3.17E-03***	**−1.83**	***0 00E + 00***
AT1G05240	Peroxidase superfamily protein	0.08	*1.00E + 00*	0.19	*1.00E + 00*	***−1.07***	***9. 14E-03***	***−1.48***	***0.00E + 00***	**−0.91**	***8 55E-07***
AT4G23400	PIP1;5_PIP1D__plasma membrane intrinsic protein 1;5	−0.29	*1.00E + 00*	−0.16	*1.00E + 00*	***−1.06***	***1.23E-02***	***−0.99***	***1.06E-07***	**−1.50**	***0 00E + 00***
AT1G69100	Eukaryotic aspartyl protease family protein	0.13	*1.00E + 00*	−0.04	*1.00E + 00*	***−1.06***	***1.39E-02***	***−1.21***	***0.00E + 00***	**−1.03**	***2 08E-09***
AT5G01210^b^	HXXXD-type acyl-transferase famiy protein	0.05	*1.00E + 00*	−0.78	*8.20E-02*	***−1.65***	***7.64E-11***	***−1.37***	***0.00E + 00***	−0.38	*1.00E + 00*
AT2G45960	ATHH2_PIP1;2_PIP1B_TMP-A__plasma membrane intrinsic protein 1B	−0.17	*1.00E + 00*	−0.11	*1.00E + 00*	***−1.14***	***1.71E-03***	***−0.77***	***2.47E-03***	−0.37	*1.00E + 00*
AT1G65930^b^	cICDH__cytosolic NADP + −dependent isocitrate dehydrogenase	−0.03	*1.00E + 00*	−0.29	*1.00E + 00*	***−1.13***	***2.23E-03***	***−0.96***	***5.06E-07***	−0.56	*1.00E + 00*
AT4G14040	EDA38_SBP2__selenium-binding protein 2	0.21	*1.00E + 00*	−0.31	*1.00E + 00*	***−1.01***	***3. 90E-02***	***−0.70***	***3.08E-02***	−0.60	*4.43E-01*
AT1G64370	unknown protein	−0.15	*1.00E + 00*	−0.22	*1.00E + 00*	***−1.01***	***3. 93E-02***	***−0.77***	***1.80E-03***	−0.16	*1.00E + 00*
***Genes differentially downregulated afteer 8 h of treatment whose levels remained low***											
AT3G03780^b^	ATMS2_MS2__methionine synthase 2	0.15	*1.00E + 00*	−0.55	*1.00E + 00*	−0.50	*1.00E + 00*	**−1.66**	***0.00E + 00***	**−2.22**	***0 00E + 00***
AT4G22210	LCR85__low-molecular-weight cysteine-rich 85	−0.16	*1.00E + 00*	−0.27	*1.00E + 00*	−0.69	*1^+00*	**−1.56**	***0.00E + 00***	**−1.27**	***0 00E + 00***
AT4G15390	HXXXD-type acyl-transferase famiy protein	0.08	*1.00E + 00*	−0.44	*1.00E + 00*	−0.70	*1.00E + 00*	**−1.39**	***0.00E + 00***	**−1.54**	***0 00E + 00***
AT3G59970^b^	MTHFR1__methylenetetrahydrofolate reductase 1	0.02	*1.00E + 00*	−0.54	*1.00E + 00*	−0.40	*1.^+0*	**−1.30**	***0.00E + 00***	**−1.06**	***2.69E-10***
AT2G44160^b^	MTHFR2__methylenetetrahydrofolate reductase 2	0.20	*1.00E + 00*	−0.28	*1.00E + 00*	−0.52	*1.00E + 00*	**−1.28**	***0.00E + 00***	**−0.95**	***1 38E-07***
AT5G39210	CRR7__chlororespiratory reduction 7	−0.09	*1.00E + 00*	0.14	*1.00E + 00*	−0.58	*1.^+0*	**−1.27**	***0.00E + 00***	**−0.89**	***2 07E-06***
AT3G19820	CBB1_DIM_DIM1_DWF1_EVE1__cell elongation protein/DWARF1 / DIMINUTO (DIM)	0.07	*1.00E + 00*	−0.11	*1.00E + 00*	−0.45	*1.00E + 00*	**−1.27**	***0.00E + 00***	**−1.15**	***0 00E + 00***
AT1G29600^a^	Zinc finger C-x8-C-x5-C-x3-H type family protein	−0.10	*1.00E + 00*	−0.15	*1.00E + 00*	−0.19	*1.00E + 00*	**−1.22**	***0.00E + 00***	**−1.02**	***2.45E-09***
AT4G12545	Bifunctional mhbitor/lpkl-transfer protein/seed storage 2S albumin superfamily protein	−0.03	*1.00E + 00*	0.28	*1.00E + 00*	−0.70	*1.00E + 00*	**−1.20**	***0.00E + 00***	**−1.66**	***0 00E + 00***
AT1G11860^b^	Glycine cleavage T-protein family	0.00	*1.00E + 00*	−0.16	*1.00E + 00*	−0.32	*1.00E + 00*	**−1.17**	***7.45E-12***	**−1.14**	***0.00E + 00***
AT3G60320	Protein of unknown function (DUF630 and DUF632)	0.03	*1.00E + 00*	−0.18	*1.00E + 00*	−0.72	*1.00E + 00*	**−1.10**	***3.72E-10***	**−0.77**	***6.14E-04***
AT5G24760	GroES-like zinc-binding dehydrogenase family protein	−0.04	*1.00E + 00*	−0.50	*1.00E + 00*	−0.34	*1.00E + 00*	**−1.08**	***1.44E-09***	**−1.19**	***0.00E + 00***
AT5G17920^b^	ATCIMS_ATMETS_ATMS1__Cobalamin-independent synthase family protein	0.13	*1.00E + 00*	−0.26	*1.00E + 00*	−0.41	*1.00E + 00*	**−1.06**	***3.43E-09***	**−0.99**	***1.51E-08***
AT3G06350b	EMB3004_MEE32__dehydroquinate dehydratase, putative/shikimate dehydrogenase, putative	−0.11	*1.00E + 00*	−0.25	*1.00E + 00*	−0.62	*1.00E + 00*	**−1.03**	***1.42E-08***	**−0.85**	***1.91E-05***
AT3G16390^b^	NSP3__nitrile specifier protein 3	0.06	*1.00E + 00*	0.20	*1.00E + 00*	0.30	*1.00E + 00*	**−1.03**	***1.88E-08***	**−1.02**	***2.19E-09***
AT1G29560^a^	Zinc finger C-x8-C-x5-C-x3-H type family protein	0.15	*1.00E + 00*	−0.38	*1.00E + 00*	−0.45	*1.00E + 00*	**−1.00**	***8.83E-08***	**−0.93**	***2.64E-07***
AT5G03300^b^	ADK2__adenosine kinase 2	0.16	*1.00E + 00*	−0.30	*1.00E + 00*	0.03	*1.00E + 00*	**−0.99**	***1.40E-07***	**−0.94**	***1.98E-07***
AT1G80830	ATNRAMP1_NRAMP1_PMIT1__natural resistance-associated macrophage protein 1	−0.02	*1.00E + 00*	−0.46	*1.00E + 00*	−0.93	*2.79E-01*	**−0.99**	***1.54E-07***	**−0.75**	***1.16E-03***
AT4G14890	FdC2__2Fe-2S ferredoxin-like superfamily protein	−0.10	*1.00E + 00*	−0.19	*1.00E + 00*	−0.48	*1.00E + 00*	**−0.97**	***2.69E-07***	**−1.01**	***4.56E-09***
AT5G65010^b^	ASN2__asparagine synthetase 2	−0.03	*1.00E + 00*	0.07	*1.00E + 00*	−0.15	*1.00E + 00*	**−0.97**	***2.79E-07***	**−0.84**	***3.11E-05***
Genes differentially downregulated only after 24 h of treatment											
AT5G36910^a^	THI2.2__thionin 2.2	−0.16	*1.00E + 00*	0.04	*1.00E + 00*	−0.38	*1.00E + 00*	0.21	*1.00E + 00*	**−1.73**	***0.00E + 00***
AT2G25510	unknown protein	−0.09	*1.00E + 00*	0.23	*1.00E + 00*	0.11	*1.00E + 00*	−0.40	*1.00E + 00*	**−1.52**	***0.00E + 00***
**AT1G17190**	**ATGSTU26_GSTU26__glutathione S-transferase tau 26**	−0.01	*1.00E + 00*	−0.19	*1.00E + 00*	0.32	*1.00E + 00*	−0.31	*1.00E + 00*	**−1.41**	***0.00E + 00***
AT3G16450	JAL33__Mannose-binding lectin superfamily protein	0.00	*1.00E + 00*	0.28	*1.00E + 00*	−0.51	*1.00E + 00*	−0.67	*9.73E-02*	**−1.30**	***0.00E + 00***
AT4G35100	PIP2;7_PIP3_PIP3A_SIMIP__plasma membrane intrinsic protein 3	0.01	*1.00E + 00*	0.10	*1.00E + 00*	−0.81	*1.00E + 00*	−0.67	*7.65E-02*	**−1.26**	***0.00E + 00***
AT3G28270	Protein ofunknown function (DUF677)	−0.26	*1.00E + 00*	0.46	*1.00E + 00*	0.25	*1.00E + 00*	−0.57	*1.00E + 00*	**−1.26**	***0.00E + 00***
AT5G51720	2 iron, 2 sulfur cluster binding	−0.06	*1.00E + 00*	−0.25	*1.00E + 00*	−0.17	*1.00E + 00*	−0.38	*1.00E + 00*	**−1.24**	***0.00E + 00***
AT5G24420^b^	PGL5__6-phosphogluconolactonase 5	0.00	*1.00E + 00*	0.05	*1.00E + 00*	−0.32	*1.00E + 00*	−0.02	*1.00E + 00*	**−1.20**	***0.00E + 00***
AT4G13870^a^	ATWEX_ATWRNEXO_WEX_WRNEXO__Werner syndrome-like exonuclease	−0.02	*1.00E + 00*	0.10	*1.00E + 00*	0.61	*1.00E + 00*	−0.34	*1.00E + 00*	**−1.19**	***0.00E + 00***
AT3G02380b	ATCOL2_COL2__CONSTANS-like 2	−0.11	*1.00E + 00*	−0.60	*1.00E + 00*	−0.41	*1.00E + 00*	0.04	*1.00E + 00*	**−1.15**	***0.00E + 00***
AT4G16980^b^	arab inogalactan-p rotein family	0.07	*1.00E + 00*	−0.10	*1.00E + 00*	−0.84	*1.00E + 00*	−0.54	*1.00E + 00*	**−1.14**	***0.00E + 00***
AT3G45140	ATLOX2_LOX2__lipoxygenase 2	−0.12	*1.00E + 00*	0.51	*1.00E + 00*	0.51	*1.00E + 00*	−0.24	*1.00E + 00*	**−1.11**	***1.50E-11***
AT1G12090	ELP__extensin-like protein	−0.06	*1.00E + 00*	−0.04	*1.00E + 00*	−0.52	*1.00E + 00*	−0.33	*1.00E + 00*	**−1.08**	***8.97E-11***
AT1G54000	GLL22__GDSL-like Lipase/Acylhydrolase superfamily protein	0.03	*1.00E + 00*	−0.28	*1.00E + 00*	−0.77	*1.00E + 00*	−0.55	*1.00E + 00*	**−1.06**	***3.59E-10***
AT5G58260^b^	NdhN__oxidoreductases, acting on NADH or NADPH, quinone or similar compound as acceptor	0.04	*1.00E + 00*	−0.01	*1.00E + 00*	−0.14	*1.00E + 00*	−0.58	*1.00E + 00*	**−1.05**	***6.51E-10***
AT3G16420	JAL30_PBP1__PYK10-binding protein 1	−0.04	*1.00E + 00*	0.07	*1.00E + 00*	0.01	*1.00E + 00*	−0.64	*2.36E-01*	**−1.02**	***2.91E-09***
AT3G16440	ATMLP-300B_MEE36_MLP-300B__myrosinase-binding protein-like protein-300B	−0.24	*1.00E + 00*	0.21	*1.00E + 00*	−0.37	*1.00E + 00*	−0.38	*1.00E + 00*	**−0.91**	***9.46E-07***
AT3G15850^b^	ADS3_FAD5_FADB_JB67__fatty acid desaturase 5	−0.21	*1.00E + 00*	−0.01	*1.00E + 00*	−0.68	*1.00E + 00*	−0.06	*1.00E + 00*	**−0.91**	***9.94E-07***
AT3G01480	ATCYP38_CYP38__cyclophilin 38	0.07	*1.00E + 00*	0.01	*1.00E + 00*	−0.76	*1.00E + 00*	−0.54	*1.00E + 00*	**−0.88**	***4.47E-06***
AT3G11170^b^	AtFAD7_FAD7_FADD__fatty acid desaturase 7	0.03	*1.00E + 00*	−0.12	*1.00E + 00*	−0.30	*1.00E + 00*	−0.63	*2.78E −01*	**−0.87**	***7 28E-06***

Of all the DEGs, a maximum of 20 genes for each different response pattern are listed. AGI identifiers and gene annotation in bold correspond to genes involved in the xenome. Expression changes are given as log2. Expression changes in bold correspond to genes differentially expressed at the significant threshold of Bonferroni *p*-value <0.05 in our study. ^a^regulatory genes. ^b^metabolic genes identified using either the MapMan pathway analysis tool choosing Metabolism-overview or the AraGEM tool [84]

### Molecular and metabolic responses during short-term PHN treatment

Considering functional classes assigned to DEGs using MapMan classification or the AraGEM tool, we found that 115 metabolic genes involved in primary and secondary metabolisms were over-represented (Additional file 2: Table S4). Nevertheless, 64 regulatory genes such as transcription factors, kinase receptors and phosphatases showed modified expression during the time course. However, in our top lists (Table 1), only 14 regulatory genes were significantly differentially expressed. Five showed the greatest change in expression after 30 min of treatment whereas all the others showed modification in expression only after 4 h of treatment. Three encoded transcription factors (*AT4G15248*, *AT4G38620 and AT5G59820*) and two encoded kinases (*AT5G48540*, *AT5G56630*) whose differential expression gradually increased over time, except for *AT4G38620*, which showed its highest differential expression at 30 min.

**Fig. 4:**
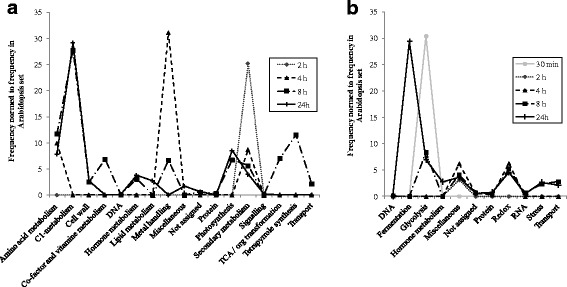
Biological pathways with significant over-representation of down- (a) and up (**b**) regulated genes (*p*-values < 0.05) after 30 min, 2, 4, 8 and 24 h of incubation in PHN-treated plants. The functional enrichment of differentially expressed genes was analyzed using the Classification SuperViewer tool from the Bio-Analytical Resource for Plant Biology (http://www.bar.utoronto.ca/ntools/cgi-bin/ntools_classification_superviewer.cgi) with MapMan classification categories. Only significant pathways are shown. Data used to generate the figure are available in Additional file 2: Table S5. The miscellaneous category corresponds to genes encoding multiple enzyme families mainly involving CYP450 and UGT in this study

We then determine specific pathways and processes significantly regulated upon PHN exposure at each time point of our kinetic analysis (Fig. 4a and b, Additional file 2: Table S5). Overall, the number of processes in which genes were significantly over-represented increased over time upon PHN exposure, with biological processes regulated after 2 h of treatment remaining induced or repressed. For upregulated genes, only glycolysis and miscellaneous metabolisms were over-represented at 30 min and 2 h of treatment, with miscellaneous and redox pathways being over-represented after 4 h of treatment. The increased number of over-represented pathways at 8 h that remain regulated at 24 h argue for a modification in plant response after 4 h of PHN exposure. These pathways include genes involved in transport, stress, RNA, redox, protein, miscellaneous, hormone, glycolysis and fermentation metabolisms. Compared to upregulated genes, genes whose steady-state expression decreased were over-represented in functional classes that mostly involve primary and secondary metabolism (cell wall, lipids, amino acids, C1, photorespiration, glycolysis/neoglucogenesis, tetrapyrrole synthesis, TCA cycle, etc.).

Although early events of sensing and signaling of xenobiotics are poorly understood, the molecular processes and metabolic pathways involved in their detoxification have been well described in animals and extrapolated to plants based on Sandermann’s green liver concept [23, 32, 33]. To identify DEGs involved in putative PHN detoxification processes, named the PHN xenome, we first recovered AGI identifiers from the TAIR website (http://www.arabidopsis.org/browse/genefamily/) of all genes that could be involved in the three detoxification phases defined by [34]. Among these genes annotated to encode cytochromes (CYP450), alpha/beta-hydrolases, glutathione S-transferase (GST), malonyl-O-transferase, sugar-dependent UDP-glycosyltransferases (UGT) and ABC transporters, 39 DEGs were identified (Additional file 2: Table S4). Their expression was primarily modified after 4 h of treatment, most of them (28/39) being differentially upregulated. In our top list (Table 1), 16 were among the most regulated genes and 15 showed upregulated expression. Except for *AT1G68620*, encoding a member of the alpha/beta-hydrolase superfamily upregulated within the first 30 min and *AT3G21560*, encoding the UDP-glycosyltransferase UGT84A2, up-regulated after 2 h of PHN exposure, most of the others (13/15) were differentially up-regulated only after 4 h. These genes mainly encode proteins belonging to GST tau (6/14) or glycosyltransferase (4/14) families.

**Fig. 5:**
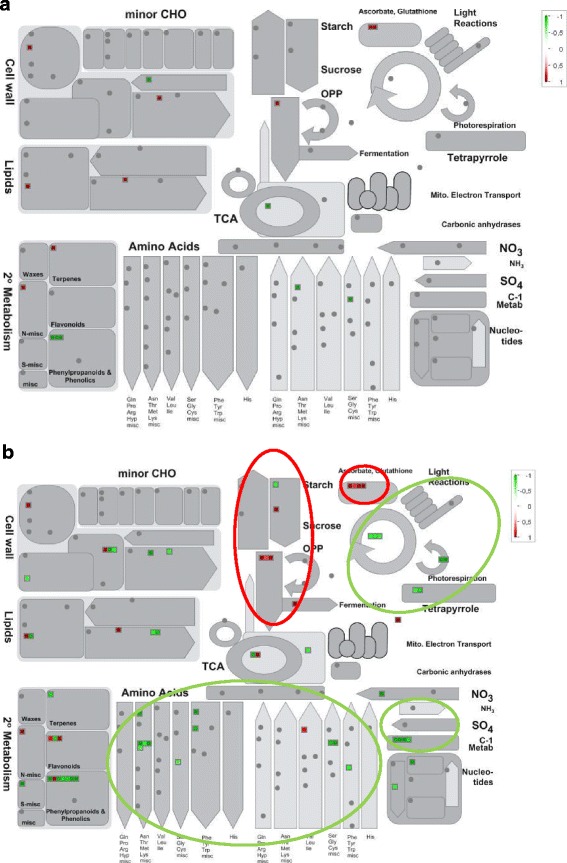
Metabolic gene expression changes at 4 h (**a**) and 8 h (**b**) of incubation with 200 μM PHN analyzed using the MapMan tool. Circles highlight major metabolic pathways in which genes are induced (*red*) or repressed (*green*). Ratios compare PHN-treated conditions to control samples

**Fig. 6:**
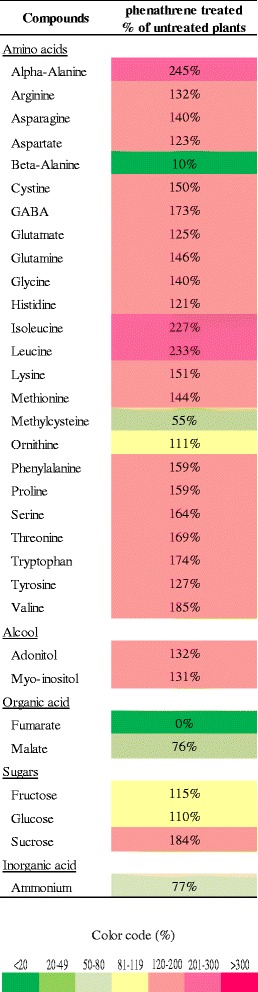
Metabolite levels in plants after 24 h of PHN treatment. Data are given as a percentage with respect to untreated plants (control). Metabolite contents were determined by GC-MS and HPLC. Data are means of three independent replicates. Citrate, galactinol, galactose, gentibiose, hydroxypiroline, maltose, mannitol, mannose, melibiose, quinate, sorbitol, succinate and trehalose were not detected in either condition

To analyze and compare gene expression changes during the experiment time-course, we used the MapMan tool for detailed visualization and comparison of individual DEGs on metabolic pathways. Figure 5 shows an example of an overview of general metabolic changes highlighted for the 4 h/8 h comparison. Lists of genes corresponding to these comparisons were chosen due to the strong differences in the number of DEGs that may reflect modification in the plant response to PHN. All the differences were also confirmed for the 24 h comparison (Additional file 1: Figure S6). From these analyses, we observed that genes involved in the Calvin cycle, photorespiration, amino acid and nucleotide synthesis were generally repressed at 8 h. Some genes involved in anaerobic metabolism, encoding a thiamine pyrophosphate-dependent pyruvate decarboxylase (*AT5G17380*), pyruvate decarboxylase 2 (PDC2, *AT5G54960*) and alcohol deshydrogenase 1 (ADH1, *AT1G77120*) proteins were upregulated (Fig. 5, Additional file 1: Figure S6). Altogether, these results suggest that photosynthetic activity is repressed, decreasing aerobic efficiency that may explain the upregulation of genes involved in glycolysis, and thus increasing the necessity for anaerobic processes. Titration of metabolites revealed that sucrose, fructose and glucose accumulated in PHN-treated plants at 24 h (Fig. 6), these changes supports the reduction of calvin cycle activity, that could be linked to less available NADPH due to reduced photosynthesis, and also correlated with upregulation of numerous known sugar-inducible genes, such as senescence-associated genes [35], observed at 24 h. Surprisingly, DEGs involved in amino-acid synthesis showed opposite trends, being downregulated while amino acids accumulated after 24 h of PHN treatment (Fig. 6). This pattern can be attributed to the induction of proteolysis, because the number of genes involved in proteolytic pathways and whose expression was upregulated at 8 and 24 h of treatment increased (Fig. 5b, Additional file 1: Figure S6). Modifications of the expression of genes identified as members of the xenome, shown in Table 1 and Additional file 2: Table S4 is also demonstrated via the visualization of the genes on biotic and abiotic overview (glutathione S-transferase) (Additional file 1: Figures S7A and S7B). This analysis also highlights the increase in genes, upregulated after 8 h and even more so after 24 h of PHN exposure, involved in plant responses to biotic and abiotic stress.

### Comparison with publicly available transcriptome data and identification of a core set of genes involved in plant response to xenobiotics

Our results were compared with the published data on the plant transcriptome response to PHN exposure (Additional file 2: Table S4, Additional file 1: Figure S8) [30]. 23 % (109/467) of our DEGs were shared, this overlap being highly significant (hypergeometric test *p*-value < 0.0001). The majority of these genes (100/109), identically regulated, showed modified expression as early as 8 h of treatment, indicating that the molecular events described in [30] are induced rapidly. These DEGs were involved in hormone, redox stress-response, metabolic pathways but also in glycolysis, fermentation processes, photosynthesis, primary and secondary metabolisms. Ten of the 15 highly regulated genes putatively involved in PHN detoxification were also common to both studies. They mostly correspond to GST, glycosyl-transferases and CYP. Strikingly, genes encoding proteins with antioxidant activity such as catalases (CAT), ascorbate peroxidase (APX) or superoxide dismutase (FSD) were not regulated in our study, whereas they were downregulated, in previous studies on Arabidopsis and tomato responses to long-term PHN exposure [30, 36].

We then compared our sets of DEGs to other transcriptome analyses performed to study the effect of various xenobiotics, such as aluminum [37], atrazine [38], benzoxazolin-2(3H)-one [39], cadmium [40], polychlorinated biphenyl [41], phenol [42] selenium [43] and trinitrotoluene [44]. Overall, 77.7 % (363/467) of DEGs were shared between the xenobiotic transcriptome datasets. Despite differences in gene regulation observed among the types of xenobiotics studied, underlining specific molecular mechanisms, we identified a core set of 363 genes, containing all 39 genes putatively involved in PHN detoxification. We propose that this core set of genes is representative of the gene network regulated in response to xenobiotics in higher plants (Additional file 2: Table S4).

Finally, we confronted our top list of DEGs (Table 1) to transcriptome dataset corresponding to the plant response to abiotic (stress selection), biotic and chemical perturbations, using the Genevestigator data base (Additional file 3: Figure S9). A hierarchical clustering analysis was performed, revealing relatively identical pattern of expression of DEGs from our top list in either abiotic (cold, drought, salt and hypoxia stress), biotic (plant response to pathogenic virus, fungi and bacteria) or chemical stress conditions (Additional file 3: Figures S9A - S9C). In the cases where opposite regulation patterns are observed in biotic stress conditions, studies correspond to comparisons in which a mutated pathogen failed to induce plant defense regulated genes [45]. As expected, this analysis performed on chemical stress experiments clustered our top list with that of the Weisman et al. [30] transcriptome data. Although most of the clustered experiments correspond to poorly described plant responses to specific pesticides and herbicides (such as Imidacloprid or sulfometuron-methyl), results indicate that detoxification processes and ROS metabolism play a major role, even in the early plant response to PHN. Our top list clustered with experiments associated with non-enzymatic lipid peroxidation by ROS (phytoprostane A1) [46], stimulation of ROS production (ozone treatment, hydrogen peroxide) experiments or effect of Fenclorim, which is known to increase the glutathione conjugation of the herbicide Pretilachlor [47].

### PHN compromises energy flow by inhibiting photosynthesis

To further explore how PHN affects plant energy transduction systems, we measured the photosynthesis and respiration rates of leaf tissues after 24 h exposure to 200 μM PHN using an oxygen electrode. Interestingly, after 24 h of treatment with PHN, leaves appeared obviously greener, which was confirmed by chlorophyll determination which revealed a 62 % increase in chlorophyll content on a fresh weight basis (Table 2). When expressed on a chlorophyll basis, PHN treatment reduced photosynthetic activity by 50 % (Table 2). When expressed on a fresh weight basis, the reduction was not statistically significant (Table 2). This result was largely due to the increase in chlorophyll content (and thus thylakoids) that likely compensates for the decrease in intrinsic photosynthetic activity. On a chlorophyll basis, respiration was not affected by PHN treatment, but it displayed a 34 % increase when expressed on a fresh weight basis (Table 2). This result indicates that the 24 h PHN treatment induced an increase in the respiratory activity of leaf tissue. There was no difference in cyanide-resistant respiration, indicating that the alternative oxidase pathway was not efficiently stimulated by the PHN treatment, although Alternative oxidase 1 a gene (*AOX1a*, *AT3G22370*) expression was significantly upregulated after 8 and 24 h of PHN treatment (Additional file 2: Table S3). Finally, the ratio between photosynthesis and respiration clearly illustrates the major impact of the short-term PHN treatment on photosynthesis (Table 2).

**Table 2:** Effect of PHN on energy metabolism after 24 h of treatment

	Control	Phenantrene	Rank sum test
	(DMSO)	200 μM	
Chlorophyll (mg.g-1 FW)	0.45 ± 0.09	0.73 ± 0.11	*P* = 0.004
Respiration with respect to chlorophyll content (nmolO2.h^−1^.mg^−1^ chlorophyll^−1^)	19.62 ± 2.52	16.11 ± 2.65	ns (*P* = 0.065)
Respiration with respect to fresh weight (nmolO2.h^−1^.mg FW^−1^)	8.74 ± 1.33	11.72 ± 2.72	*P* = 0.041
Cyanide-resistant respiration (%)	46.86 ± 5.07	48.70 ± 7.6	ns (*P* = 0.485)
Photosynthesis with respect to chlorophyll content (nmolO2.h^−1^.mg^−1^ chlorophyll^−1^)	79.11 ± 5.96	40.56 ± 6.62	*P* = 0.004
Photosynthesis with respect to fresh weight (nmolO2.h^−1^.mg FW^−1^)	35.93 ± 6.31	29.46 ± 6.46	ns (*P* = 0.082)
Photosynthesis/respiration ratio	4.10 ± 0.34	2.54 ± 0.35	*P* = 0.004

Four-week-old plants grown in vitro in MS/2 medium were treated for 24 h with 200 μM PHN before measurements of leaf chlorophyll content, respiration and photosynthesis. Data are indicated as average with SD (*n* = 5 or 6), and due to the small sample size, a non-parametric Mann–Whitney rank sum test was applied (*ns* non significant)

**Fig. 7:**
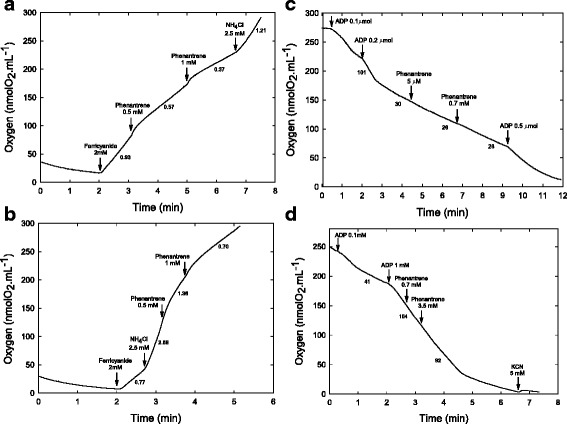
Effect of PHN on isolated mitochondria and thylakoids. The impact of PHN was analyzed using isolated spinach thylakoids (**a**, **b**) or pea seed mitochondria (**c**, **d**). For thylakoids, the light-driven oxygen production with ferricyanide as a photosystem I acceptor was measured using well-coupled thylakoids (**a**) or thylakoids uncoupled by by NH_4_Cl (**b**). The arrows indicate the addition of the different compounds (final concentration) and the number below the line refers to the corresponding rate (μmol O_2_.mg chlorophll^−1^.min^−1^). For pea seed mitochondria, PHN was added to isolated organelles oxidizing 5 mM succinate in state 4 (**c**) or state 3 (**d**). Arrows show the addition of compounds with their final concentration or amount in the case of ADP. Numbers under the lines indicate the rate of oxygen consumption in nmolO_2_.min^−1^.mg prot^−1^

**Fig. 8:**
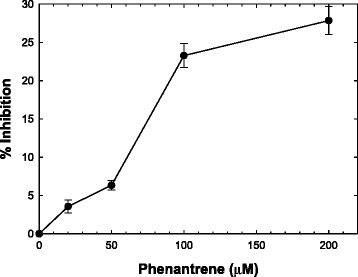
Inhibitory effect of PHN at low concentrations on thylakoid electron transfer. The impact of PHN on thylakoid electron transfer was measured using light-driven oxygen production with ferricyanide as a photosystem I acceptor on well-coupled thylakoids. Experiments were done in triplicate and SD is indicated

To explore further the impact of PHN, its direct effects on energy-transducing organelles were studied using mitochondria isolated from imbibed pea seeds and chloroplasts isolated from spinach leaves. Even at high concentrations, PHN had no uncoupling effect on the electron transfer in mitochondria or thylakoids. Although PHN is highly lipophilic, it is not protonable and does not seem to interfere with proton permeability of the membrane (Fig. 7a and b). However, PHN in the mM range was found to strongly inhibit electron transfer in thylakoids, but not in mitochondria (Fig. 7a, c and d). Dose–response analysis showed that inhibition of thylakoid electron transfer could be detected even at 20 μM (Fig. 8). Since such effects are detected within a few minutes after the addition of the compound, it is thus likely that a low dose of PHN accumulating in leaf cells can affect photosynthesis, leading to progressive exhaustion of plants. Interestingly, mitochondrial respiration was not affected by PHN. Hence, the increased leaf respiration that occurs after 24 h of treatment could reflects a higher energy demand in response to stress, possibly including biogenesis of thylakoids associated with chlorophyll biosynthesis.

## Discussion

Our results show that PHN alters plant development in a concentration-dependent manner, in accordance with previous data described in Alkio et al. [27] and Liu et al. [31]. However, our conditions (i.e. sucrose-free medium) showed major differences from the previous studies, such as chlorotic phenotypes at higher concentrations, absence of hypersensitive response-like necrosis on leaves and phenotypic heterogeneity within a given treatment. Hence, the use of sucrose-free medium appears to reveal the true phenotype induced by PHN exposure. Many studies investigating sugar signaling networks indicate that sugars play a crucial role in plant development [48] and emerged as efficient ROS scavengers in plants, thereby limiting ROS production in stress conditions. Furthermore, sucrose availability can enhance phytoaccumulation of organic pollutants such as atrazine [49].

Root absorption of PHN occurs via passive diffusion and an active PHN uptake involving a -specific H+/PHN symporter [29]. Surprisingly, we did not detect any PHN fluorescence in roots. One explanation is that PHN or its derivatives are rapidly transported as conjugates through the xylem to leaves. The majority of organic contaminants absorbed by plants undergo enzymatic transformation that reduce their toxicity and allow their conjugation [33] These processes may also modify their UV absorbance and fluorescence. For example, PHN derivatives, metabolized by the white rot fungus *Phanerochaete chrysosporium*, have different UV absorption spectra [50], making them more difficult to detect, except in specific locations where they aggregate and are compartmentalized. However, our results suggest that PHN is absorbed, at least partially from roots, transported and accumulates in trichomes or volatilized through stomata as suggested by [51]. Alternatively, we cannot rule out that this may also be an artifact of using DMSO as solvent of the PHN, since the roots are in contact with DMSO in the medium. As seems to be the case for PHN or its derivatives in our study, several lines of evidence suggest that trichomes participate in heavy metal detoxification through the formation of metal/calcium crystals actively excreted in *Nicotiana tabaccum* [52, 53] or the accumulation of zinc and cadmium in a specific subcellular compartment at the base of trichomes *Arabidopsis halleri* [54]. Moreover, the characterization of mutants for stomatal aperture may help to determine the role of stomata in excretion.

The dissection of the early plant response to PHN exposure adds a new level of resolution to previous studies [30]. Based on our experimental design, the proportion of DEGs shared with the long-term exposure study [30] was rather low except for our 24 h time point and a high proportion of early response specific DEGs were revealed. Thus, these new DEGs pave the way to the characterization of new actors that can be engineered to improve PHN and PAH phytoremediation. Moreover, the significant overlap of DEGs indicates that antioxidant or detoxification processes, repression of photorespiration and shift from anabolism to catabolism are unexpectedly quickly set up. This rapid response to PHN unravels mechanisms deployed to cope with PHN injuries. Furthermore, comparisons with previous studies on other xenobiotics [37, 38, 40–44] identified a core set of 363 genes, suggesting that plant response to xenobiotics relies on the regulation of similar gene networks, probably induced in response to secondary entities also produced in other biotic or abiotic stress conditions (e.g. see [55]).

**Fig. 9:**
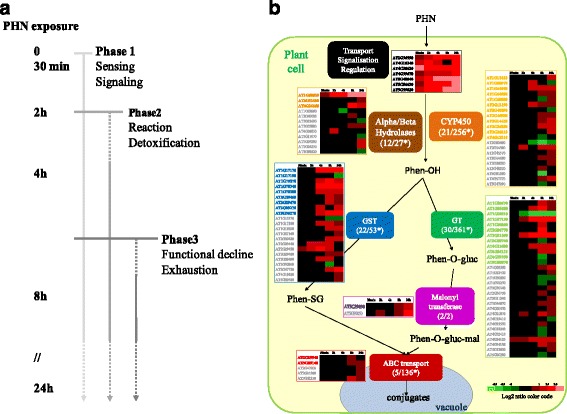
Proposed model of the early plant response to PHN exposure. **a** Kinetic representation of the early plant response to PHN. Following sensing and signaling of PHN within the first 30 min (phase 1), Arabidopsis induces the expression of genes involved in the detoxification and reaction response to PHN, from 2 to 24 h (phase 2). After 8 h of incubation, the regulation of the expression of numerous genes involved in primary and secondary metabolisms, as well as specific primary metabolite accumulation suggest functional declines (phase 3). **b** Identification of the genes assumed to be involved in sensing, signaling and the xenome according to the model described in [24]. Numbers indicate the number of genes belonging to each family, and that were differentially expressed in our study, compared with the total number of genes classified in each family. *: gene members of each family were retrieved from the TAIR website (http://www.arabidopsis.org/browse/genefamily/). Genes indicated in bold in each family correspond to differentially expressed genes selected after ANOVA analysis

This study revealed rapid changes in gene expression within the first 24 h of PHN exposure that might correspond to an adaptive strategy developed by plants to sense xenobiotics and activate molecular processes devoted to PHN detoxification. This early plant response to PHN seems to follow the Larcher model [56]. In the model presented in Fig. 9, we propose that the plant response is divided into three phases (Fig. 9a).

1. Within the first 30 min of exposure (first phase), only 14 genes are rapidly expressed and continuously upregulated during the time course. Among them are regulatory genes encoding a receptor-like kinase protein (RLK) (*AT5G48540*), and three transcription factors that may participate in sensing or signaling of PHN. Interestingly, *ATZAT12* (*AT5G59820*) has been described to play a central role in light acclimatization and plant responses to cold and oxidative stress [57–59]. Increased expression of *ATMYB4* (*AT4G38620*) has also been observed in response to cadmium exposure [60], confirming that transcription factors are important regulators of plant responses to stress. Plant alpha/beta-hydrolases, are proteins that can hydrolyze xenobiotics in phase I of the xenome [34]. The fast upregulation of a gene (*AT1G68620*) encoding such proteins 30 min after PHN treatment suggests that this gene is of particular interest for future developments in PHN phytoremediation. Intriguingly, the fast upregulation of *ELIP1*, encoding an early light-induced protein known to bind chlorophyll and play a role in photoprotection [61], suggests that chloroplast functions are rapidly affected by PHN. This assumption is strongly supported by our analysis showing that even low doses of PHN in leaves inhibit photosynthesis within a few minutes. These results are in accordance with the photosynthesis inhibition effects of 2 weeks PHN exposure on several crops [62] and physiological responses to early abiotic stress often characterized by reduced photosynthetic efficiency [63]. The strong and early inhibition of electron transfer in thylakoids may rapidly reduce energy production, increase energy demand and destabilize ROS homeostasis. Continuous upregulation of *PFK7*, which encodes the phosphofructokinase 7, a major regulator of glycolysis pathway, corroborates a rapid and strong demand for energy and/or reductants within the first minute to withstand PHN exposure. The rapid mRNA accumulation of *ELIP1* and *At4g39670*, an H_2_O_2_-induced gene [64], also supports this hypothesis.
2. A reaction phase corresponding to processes induced by plants to detoxify PHN and build a stress response, from 30 min up to 4 h of treatment. We identified 39 DEG genes classified in the PHN xenome, all regulated at 24 h. The 13 genes upregulated as early as 4 h following PHN exposure suggest that detoxification processes are engaged just a few hours after treatment. They mostly correspond to CYP involved in putative transformation of PHN (phase 1), UGT and GST of the tau family known to catalyze the conjugation and detoxification of numerous xenobiotics (phase 2). Gene ontology enrichment analysis and comparisons with other publicly available data provide clear evidence that detoxification processes occur, but also that ROS are rapidly produced. An imbalance in ROS and redox may effectively originate from PHN-driven inhibition of photosynthesis. ROS are known to cause cytotoxic damage on photosynthetic membranes and specifically activate programs to alleviate the effect of environmental stress or to trigger cell death [65, 66]. Surprisingly, antioxidant genes such as *CAT*, *FSD1* and *APX*, previously found to be regulated during long-term exposure to PHN [30], were not differentially regulated in our study. However, upregulation of *ATGSTU8*, *UGT74E2* and *UGT73B3*, between 2 and 8 h of PHN exposure, indicates that the plant tries to minimize the impact of ROS overproduction. Indeed, ATGSTU8 has been suggested to counteract the effect of high ROS production in stressed plants [67]. On the other hand, UGT74E2 may allow integration of ROS through its activity on auxin indole-3-butyric acid [68] and UGT73B3 participates in the regulation of redox status and general detoxification of ROS during the hypersensitive response triggered by the avirulent bacteria *Pseudomonas syringae pv. tomato* (Pst-avrRpm1) [69].
3. The increased respiratory activity detected in leaves after 24 h of treatment provides further evidence supporting the assumption of a higher energy demand. Due to the reduction in photosynthesis efficiency, it is not surprising to observe a significant over-representation of induced genes involved in both glycolysis and fermentation after 8 h of treatment, probably to sustain production of ATP and reductants, such as NADH/NADPH. Furthermore, plant responses to early stress not only affect photosynthetic activity but also repress transport metabolism and induce accumulation of metabolites and/or uptake-translocation of ions [63]. The strong transcriptome reprogramming and the shift of several metabolic pathways to catabolism result in the accumulation of amino acids and soluble sugars, indicators of functional decline. The observed decline in photosynthesis associated with downregulation of genes involved in photosynthesis after 8 h of treatment corroborate the idea that the carbon/nitrogen balance and photosynthetic activity are inextricably linked through feedforward and feedback regulation mechanisms [70]. Thus, PHN inhibition of photosynthetic energy transduction may be the early crucial event that leads to the third phase of our model: the impending exhaustion of the plant [30].

## Conclusion

Altogether, our results highlight the rapid impact of PHN on photosynthesis that highly imbalanced energy flow and progressively leads to plant death. The identification of molecular events involved in the early plant response to PHN reveals primary processes set up to sense and detoxify PHN that are potential targets for improvement and engineering future phytoremediation strategies. 39 specific genes identified as members of the PHN xenome correspond to proteins performing transformation (CYP alpha/beta-hydrolases; phase 1), the conjugation of xenobiotics (malonyl-O-transferase, GST, UGT; Phase2) and the compartmentalization of conjugated products in the vacuole or the cell wall (ABC transporters; phase 3). They are the most relevant candidates that could be involved in the PHN detoxification and will be considered for subsequent studies using reverse genetic approaches.

## Methods

### Plant material and growth conditions

Seeds of *Arabidopsis thaliana* accession Columbia-0 (Col-0) were surface-sterilized and sown on half-strength Murashige and Skoog (MS/2) solid medium containing 0.8 % (w/v) agar-agar type E (Sigma Aldrich) supplemented with PHN dissolved in dimethylsulfoxide (DMSO) or DMSO alone used as control. Seeds were germinated in a growth chamber (16:8 h light:dark cycle, 4000 lux, 22 °C, 70 % hygrometry) after cold treatment for 48 h at 4 °C.

### Measurement of seedling growth and development

Seeds were sown on MS/2 solid medium supplemented with 0, 50, 100, 200 and 400 μM of PHN or with DMSO alone as a control. For primary root length measurements, plants were cultivated vertically on square Petri dishes (15 × 15 cm). Primary roots were measured after 10 days of growth using digital photographs analyzed using Image J v 1.45 s software [71]. For each condition, primary root length of at least 18 independent plants was measured. Rosette fresh weight and chlorophyll content measurements were carried out as described in [51]. Results are given as the mean of six measurements for the four independent biological replicates. Statistical analyses were carried on using the *t*-test in R [72].

### PHN quantification

Plants used for PHN quantification were grown for 15 days on MS/2 and then transferred to liquid medium containing 200 μM PHN. After 24 h of incubation, plants were harvested and rinsed with water, absolute ethanol and again with water. Plant samples were dried, then ground and weighed. PHN was extracted from three independent samples composed of pooled plantlets, using an accelerated solvent extractor (ASE 200, Dionex) with dichloromethane at 100 °C and under a pressure of 100 bars. Extracts were dried under a gentle flow of nitrogen gas in a pre-weighed vial and weighed to determine the mass of extract. Extract were diluted with dichloromethane (4 mg/ml). Then, 1 μL of the extract was injected onto a Shimadzu QP2010 + MS gas chromatograph/mass spectrometer (GC-MS) (Shimadzu, Tokyo, Japan). The injector used was in splitless mode and maintained at a temperature of 310 °C. The gas chromatographic separation was performed on a fused silica SLB-5 ms capillary column (from Supelco; length, 60 m; diameter, 0.25 mm; film thickness, 0.25 μm) under the following temperature program: 70 °C (held for 1 min) to 130 °C at a rate of 15 °C/min, then 130 °C to 300 °C (held for 15 min) at a rate of 3 °C/min. Helium flow was maintained at 1 mL/min. The chromatograph was coupled to the MS by a transfer line heated to 250 °C. Analyses were performed in selective ion monitoring mode. The mass spectrum was scanned from 50 to 600 nm in the Electron Ionization (EI) mode (70 eV), using the quantifier ions 178. Quantification was based on the internal standard PHN-d10, which was added to the sample post-extraction and prior to the GC-MS analysis.

### Fluorescence microscopy

Arabidopsis plantlets were grown on MS/2 medium for 15 days and then transferred at the four rosette-leaf stage (stage 1.04) (Boyes et al., [73]) to MS/2 medium containing 200 μM PHN or the same volume of DMSO. Plants were grown vertically. A sterile transparent plastic film was applied to the contaminated medium to avoid any contact of the vegetative parts of the plantlets with the medium. After 5 days of treatment, leaves and roots from five independent plantlets harvested from three different Petri dishes and were observed either with a Zeiss Axioplan Imaging epifluorescence microscope using the DAPI filter cube (Ex 365/50-D 395-Em 445/50) or a Leica SP-2 AOBS confocal microscope using a 405 nm diode laser as the excitation source. Specific PHN fluorescence emission was collected in the 420–460 nm range, cell autofluorescence was collected in the 530–580 nm range and chlorophyll emission in 660–700 nm range.

### Analysis of respiration and photosynthesis

Oxygen consumption and its variation over time were measured with a liquid-phase Oxytherm oxygen electrode system (Hansatech) as described in [74], except analysis was done on fragmented leaves. Leaves were cut into pieces with a scalpel and vacuum infiltrated with 4 mM KH_2_PO_4_, pH 6.8, 10 mM sucrose. Plant material was recovered by filtration on a 46 μm nylon mesh and around 60–80 mg (fresh weight) was immersed in 1 mL of the same medium, supplemented with 0.5 mM NaHCO_3_, in the Oxygraph (Hansatech) measurement chamber. Analysis was done in the dark under continuous stirring at 25 °C. The system was operated at 25 °C, at maximum stirring speed, and respiratory oxygen consumption was measured in the dark, and photosynthetic oxygen production was measured upon illumination using an optic fiber illuminating system providing 700 μmol.m^−2^.s^−1^. A complex IV inhibitor, potassium cyanide, and an inhibitor of AOX, n-propylgallate, were injected later during the course of the experiment. To measure fresh weight and chlorophyll content, the leaf fragments were recovered by filtration as above, weighed and incubated at 4 °C in the dark in 1 mL N,N-dimethylformamide to extract chlorophyll, which was then quantified spectrophotometrically in a multi-well quartz plate using a spectrophotometer (Fluostar Omega, BMG LABTECH GmbH, Ortenberg, Germany). Total chlorophyll (Ct = 7.04 A_664_ + 20.27A_647_, in μg.ml^−1^) was calculated according to [75].

#### Organelle isolation, functional analysis

The isolation of intact pea seed mitochondria and functional analyses of them using oxygraphy are described in [76]. The effect of various concentrations of PHN was studied on mitochondria oxidizing different substrates (5 mM succinate, 3.75 mM malate + 3.75 mM glutamate + 2 mM pyruvate, 1.5 mM NADH), using an Oxytherm respiration measurement system (Hansatech) in the absence or presence of ADP, using appropriate cofactors when necessary [76].

#### Thylakoid membranes isolation

Spinach leaves obtained from a local market were used to isolate chloroplasts according to a classic procedure [77]. Class C chloroplasts corresponding to well-coupled thylakoid membranes were used to monitor light-dependent oxygen production with ferricyanide as an acceptor, and NH_4_Cl as an uncoupler when needed. Oxygen production was monitored with a DW1 Oxygraph (Hansatech) in a medium containing 330 mM sorbitol, 4 mM MgCl2, 10 mM HEPES (4-(2-hydroxyethyl)-1-piperazineethanesulfonic acid) pH 7.8.

### Transcriptome studies

Microarray analysis was carried out at the Research Unit in Plant Genomics in Evry, France, using the CATMA version 5 array containing 31,776 gene-specific tags corresponding to 22,089 genes from Arabidopsis [78, 79]. Total RNA extractions from two independent biological replicates were performed using the Qiagen RNAeasy plant minikit according to the manufacturer’s instructions. Each biological replicate was composed of Arabidopsis plantlets grown in vitro for 15 days on solid MS/2 medium and transferred at stage 1.04 [73] to liquid MS/2 medium containing 200 μM PHN or the same volume of DMSO. Each biological replicate included PHN-treated and control plants. Each sample consisted of 30 plants that were pooled and harvested after 30 min, 2, 4, 8 or 24 h of incubation. For all conditions (Additional file 1: Figure S1A), the experiment was done using the dye-switch technique. The labeling of antisense-amplified RNA with Cy3-dUTP or Cy5-dUTP (Perkin-Elmer-NEN Life Science Products), hybridization to slides, and scans were performed as described in [80].

#### Statistical analysis of microarray data

Statistical analyses were carried out as described in [81]. The complete data set is given in Additional file 2: Table S2. For ANOVA analysis, normalized intensities for each dye-switch experiment were recovered. A sample is characterized by the time-point of the experiment (30 min, 2, 4, 8 or 24 h), the treatment (DMSO or PHN), the dye used for the experiment (red or green) and the array on which the sample was hybridized (numbered from 1 to 28). For a given gene, we denoted Y_*tpda*_ the expression level of the gene at time-point *t*, with treatment *p*, using dye *d* and on array *a*. We studied two linear models. The first (Model 1) considered an additive effect of time (α_*t*_) and treatment (β_*p*_) without interaction. The second model (Model 2) considered an additive effect of time (α_*t*_) and treatment (β_*p*_) and an interaction between the two (γ_*tp*_). In both models, a potential array effect (δ_*a*_) was included. We only analyzed genes for which all 56 data points were available, i.e. genes without missing values.

Model1 $$ {\mathrm{Y}}_{tpda}=\upmu +{\upalpha}_t+{\upbeta}_p+{\updelta}_a+{\upvarepsilon}_{tpda} $$

Model2 $$ {\mathrm{Y}}_{tpda}=\upmu +{\upalpha}_t+{\upbeta}_p+{\upgamma}_{tp}+{\updelta}_a+{\upvarepsilon}_{tpda} $$

For each gene, the parameters of Model 1 and 2 were fitted using ordinary least squares. Model 1 had 22 residual degrees of freedom and model 2, only 18. For each gene, we used a Fisher test to test the hypothesis that Model 1 is true, against the alternative hypothesis that Model 2 is true. We accounted for multiple testing using the Benjamini-Hochberg procedure. We considered that genes with an adjusted *p*-value of less than 5 % showed an interaction between time and treatment. All these analyses were performed with R software [72]. Data corresponding to selected genes are presented in Additional file 2: Table S3.

### Venn diagrams and biological pathway enrichment

Lists of genes, considered to have a time-treatment interaction (adjusted ANOVA *p*-value < 5 %), were recovered for comparisons of 30 min, 2-, 4-, 8-, and 24 h time points. Venn diagrams were generated using the Venn SuperSelector tool whereas biological pathways significantly over-represented in lists of DEGs were identified with the Classification SuperViewer tool on the University of Toronto website (http://bar.utoronto.ca/ntools/cgi-bin/ntools_classification_superviewer.cgi) using MapMan classification categories [82]. For Venn diagrams, significance of the overlap between downregulated or upregulated genes lists compared was assessed using an exact Fisher test.

### Clustering

Hierarchical clustering analyses were performed via the Genevestigator toolbox for plant biology (https://www.genevestigator.com/gv/), with our top list (Table 1) measured as Euclidian distance, and based on stress, biotic and chemical data. Data were selected by sample, filtering on wild type genetic background. The stress, biotic and chemical selections correspond respectively to 788 samples from 310 perturbations, 450 samples from 111 perturbations and 500 samples from 117 perturbations.

### Targeted analysis of metabolites

Analyses were carried out at the CORSAIRE platform (Biogenouest, INRA UMR 1349 IGEPP, Le Rheu, France). Arabidopsis plants used were grown on MS/2 medium for 15 days and then transferred at stage 1.04 [73] to liquid MS/2 medium containing 200 μM PHN or the same volume of DMSO. After 24 h incubation, plants were harvested, frozen in liquid nitrogen, lyophilized and ground. A total of 10 mg of dry plant material was used. Extraction, amino acid, sugar, organic acid, alcohol and ammonium quantification were carried as described by [83].

## Additional files

Additional file 1: Figure S1. Emission spectra of PHN in different solutions. **Figure S2.** Confocal observations and spectra of PHN in its crystal state (A, B, C) or as aggregates on leaf (D, E, F). **Figure S3.** Representative confocal microscope projections realized on a PHN aggregate observed on the epidermis on the abaxial side of the third leaf of in 20-day-old plantlet. **Figure S4.** **A** Schematic representation of the experimental procedure for transcriptome profiling of the Arabidopsis response to PHN. **Figure S5.** QPCR validations of transcriptomic data. **Figure S6.**: Overview of the metabolic gene expression changes after 24 h of 200 μM PHN treatment, analyzed by the MapMan tool. **Figure S7**: Biotic stress gene expression changes after 8 h (**A**) and 24 h (**B**) of incubation with 200 μM PHN, analyzed using the MapMan tool. **Figure S8.** Number of differentially expressed genes (DEGs) found specifically in our study or in Weisman et al. [30], and shared between both studies. (PDF 1821 kb)
Additional file 2: Table S1. Transcriptome data obtained for each performed comparison. **Table S2.** Genes found to be differentially expressed in all the comparisons between PHN-treated and control plants (hybridizations 5–9). **Table S3.** Genes found to be differentially expressed in all the comparisons between PHN-treated and control plants (hybridizations 5–9), selected after ANOVA analysis. **Table S4.** The 467 DEGs found to be differentially expressed in all the comparisons between PHN-treated and control plants (hybridizations 5–9), selected after ANOVA analysis. **Table S5.** Biological pathways with significantly over-represented genes. **Table S6.** Primers used for quantitative RT-PCR. (XLSX 17507 kb)
Additional file 3: Figure S9. Hierachical clustering analysis within Genevestigator public data. (PDF 698 kb)

## Abbreviations

ADH1: Alcohol deshydrogenase 1
AOX1a: Alternative oxydase 1 a
APX: Ascorbate peroxidase
CAT: Catalase
Col-0: Arabidopsis accession Columbia-0
CYP: Cytochrome
DEGs: Differentially expressed genes
DMSO: Dimethylsulfoxide
ELIP1: Early light-induced protein 1
FSD: Superoxide dismutase
GC-MS: Gas chromatography mass spectrography
GST: Glutathione s-transferase
HPLC: High performance liquid chromatography
MS/2: Half-strength Murashige and Skoog medium
PAHs: Polycyclic aromatic hydrocarbons
PDC2: Pyruvate decarboxylase 2
PFK7: Phosphofructokinase 7
PHN: Phenanthrene
Pst: Pseudomonas syringae pv. tomato
RLK: Receptor-like kinase
ROS: Reactive oxygen species
TCA: Tricarboxylic acid
UGT: UDP-glycosyltransferase
