# Supplementary material for: Unraveling the early molecular and physiological mechanisms involved in response to phenanthrene exposure
Source: BMC Genomics. 2016 Oct 21;17:818. doi: 10.1186/s12864-016-3133-0 (PMC5073745; doi:10.1186/s12864-016-3133-0)
Supplement: Additional file 1: Figure S1. — Emission spectra of PHN in different solutions. Figure S2. Confocal observations and spectra of PHN in its crystal state (A, B, C) or as aggregates on leaf (D, E, F). Figure S3. Representative confocal microscope projections realized on a PHN aggregate observed on the epidermis on the abaxial side of the third leaf of in 20-day-old plantlet. Figure S4. A Schematic representation of the experimental procedure for transcriptome profiling of the Arabidopsis response to PHN. Figure S5. QPCR validations of transcriptomic data. Figure S6.: Overview of the metabolic gene expression changes after 24 h of 200 μM PHN treatment, analyzed by the MapMan tool. Figure S7: Biotic stress gene expression changes after 8 h (A) and 24 h (B) of incubation with 200 μM PHN, analyzed using the MapMan tool. Figure S8. Number of differentially expressed genes (DEGs) found specifically in our study or in Weisman et al. [30], and shared between both studies. (PDF 1821 kb) [file 12864_2016_3133_MOESM1_ESM.pdf]

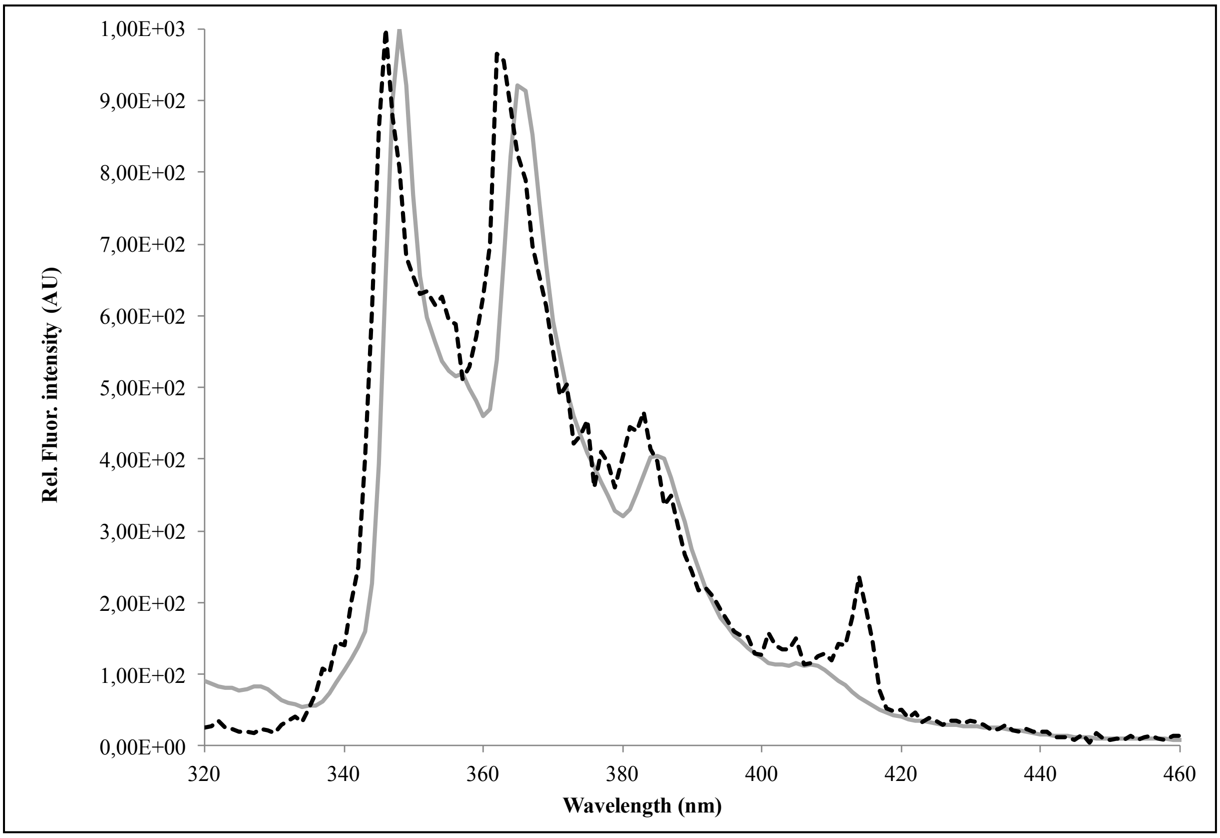


**Figure S1:** Emission spectra of PHN in different solutions. Continuous grey line: PHN in DMSO (2.3 nM). Dashed black line : PHN disolved in DMSO, then diluted in water (2.3 nM).

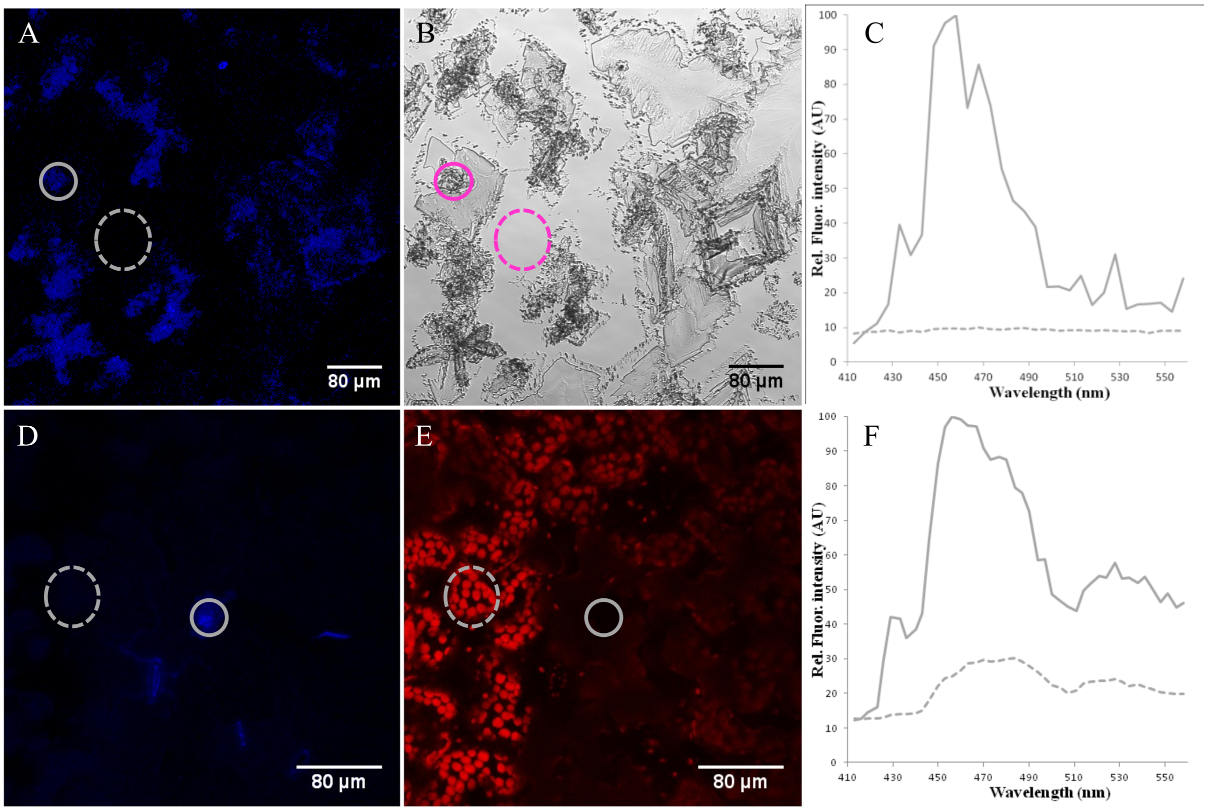


**Figure S2:** Confocal observations and spectra of PHN in its crystal state (A, B, C) or as aggregates on leaf (D, E, F).

Excitation at 250 nm provided emission lines from which PHN could be specifically detected, even when a 405 nm excitation wavelength was used. Then, using a 405 nm UV laser and a confocal microscope, PHN produced two major emission lines at 430 nm and 455 nm in crystals and leaves. A: confocal optical section of PHN crystals obtained after evaporation of 200 µM PHN solution. B: corresponding bright field observation. Confocal optical section of the third leaf of in 20-day-old plantlet showing PHN aggregates on its abaxial side (D, C). D: Specific PHN emission (blue) was collected in the 420-460 nm range using a 405 nm diode laser as the excitation source. E: Chlorophyll emission (red) was collected in 660-700 nm range. C, D: Specific PHN spectra (continuous grey lines) obtained from the areas indicated by continuous circles. Dashed grey lines correspond to spectra obtained from the areas without specific PHN emission, indicated by dashed circles.

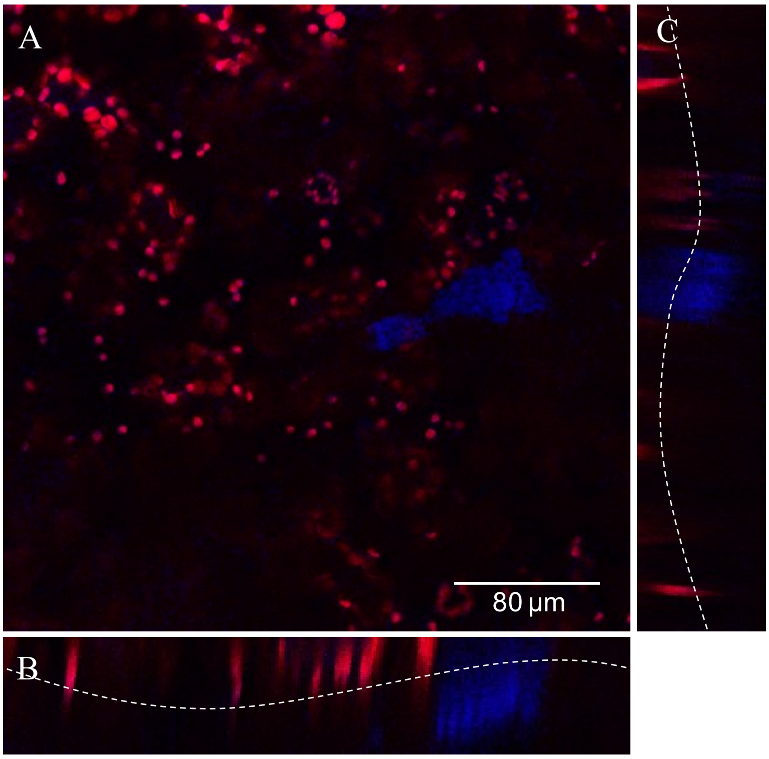


**Figure S3:** Representative confocal microscope projections realized on a PHN aggregate observed on the epidermis on the abaxial side of the third leaf of in 20-day-old plantlet.
A-C: panels showing XYZ (A), XZ (B) and YZ (C) projections. Dashed grey lines indicate epidermis surface (B, C). Specific PHN fluorescence emission (blue) was collected in the 420-460 nm range with a Leica SP-2 AOBS confocal microscope using a 405 nm diode laser as the excitation source. Chlorophyll emission (red) was collected in 660-700 nm range.

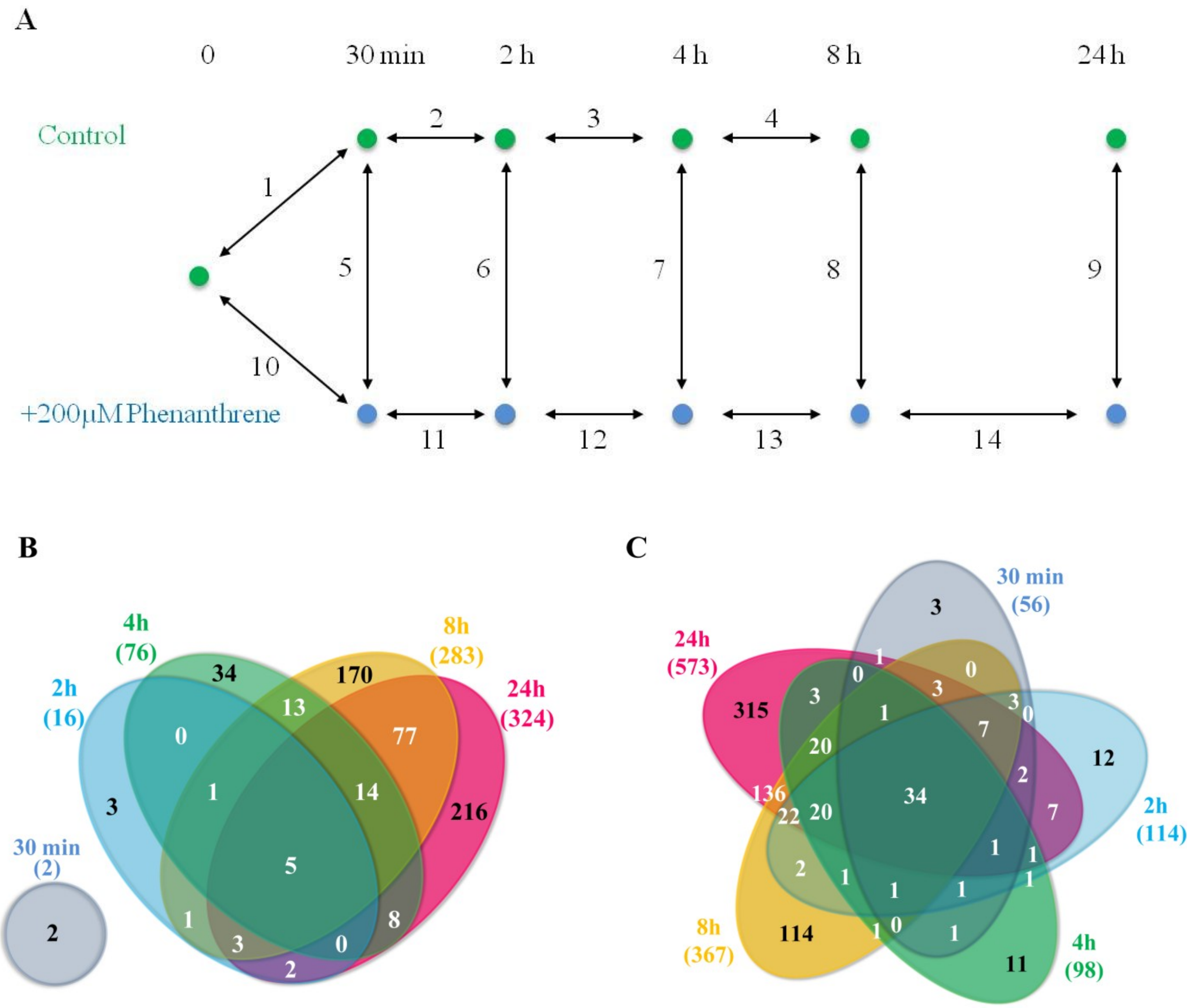


**Figure S4: A:** Schematic representation of the experimental procedure for transcriptome profiling of the Arabidopsis response to PHN.

Fourteen comparisons (1–14) of control and treated plants with 200 µM PHN were performed at five time points (30 min, 2 h, 4 h, 8 h and 24 h). Double arrows indicate the dye-switch hybridization described in Materials and Methods for the two biological replicates used. In comparisons 1-4, RNAs from control plants from conditions 0, 30 min, 2 h and 4 h were hybridized on the same array with RNAs from control plants in the same condition at 30 min, 2 h, 4 h, 8 h respectively. In comparisons 5-9, RNAs from control plants at 30 min, 2 h, 4 h, 8 h and 24 h were hybridized with RNAs from PHN-treated plants corresponding to the same time points. In comparisons 10-14, RNAs from PHN-treated plants at 0, 30 min, 2 h, 4 h and 8 h were hybridized on the same array with RNAs from PHN-treated plants at the 30 min, 2 h, 4 h, 8 h and 24 h time points, respectively. (**B**, **C**) Venn diagrams showing the total number of DEG (p-value $< 0.05$). Lists of genes correspond to down-regulated (**B**) and up-regulated genes (**C**) obtained by comparing PHN treated samples with control samples at 30 minutes, 2h, 4h, 8h and 24h. DEG used are listed in Table S2. Transcriptome data corresponding to the performed hybridizations are available in Table S1.

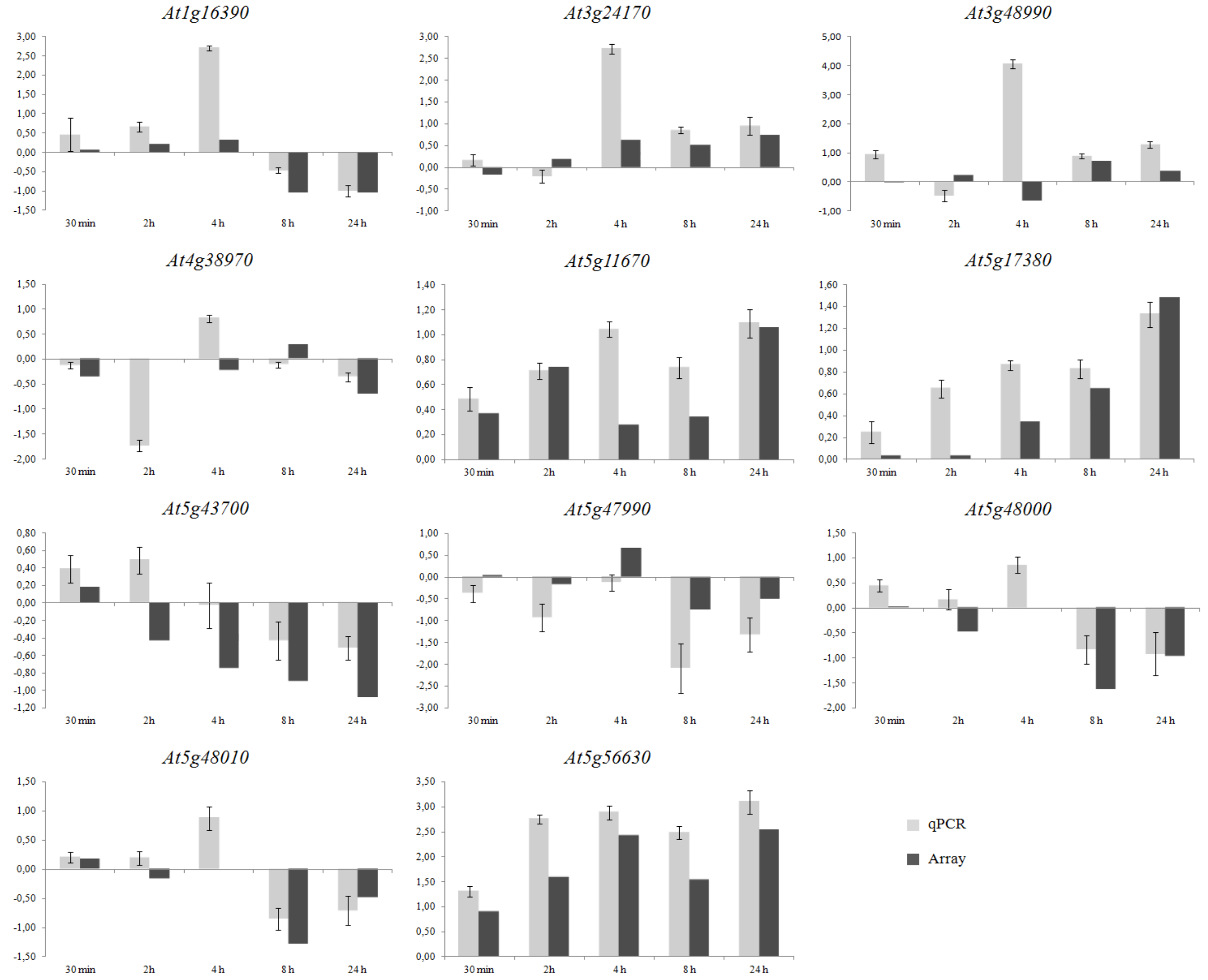


**Figure S5:** QPCR validations of transcriptomic data**.**

The $\log_2$ ratios from CATMA microarray experiments and subsequent validation by qPCR ($\Delta C_t$) samples for 30 min, 2 h, 4 h, 8 h and 24 h comparisons for 11 selected transcripts. QPCR $\Delta C_t$ values are means ±SE of three replicates. Normalized ΔCt values for each differentially expressed gene were calculated as follows: Norm ΔCt =-(( $C_{t1}$-$C_{t2}$)-NF) where NF is the average normalization factor calculated on the basis of results obtained for two best control genes chosen for their consistent expression in CATMA microarray experiments in all samples (*At4g13615*, *At5g21090*).

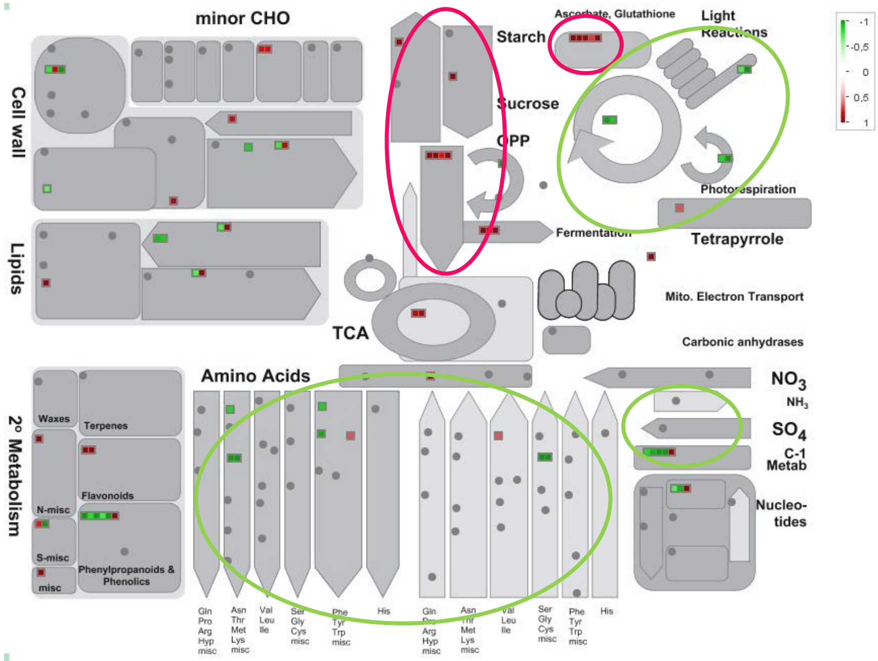


**Figure S6:** Overview of the metabolic gene expression changes after 24 h of 200 µM PHN treatment, analyzed by the MapMan tool.
Circles highlight major metabolisms in which genes are induced (red) or repressed (green). Ratios compare PHN treated samples to control samples.

B

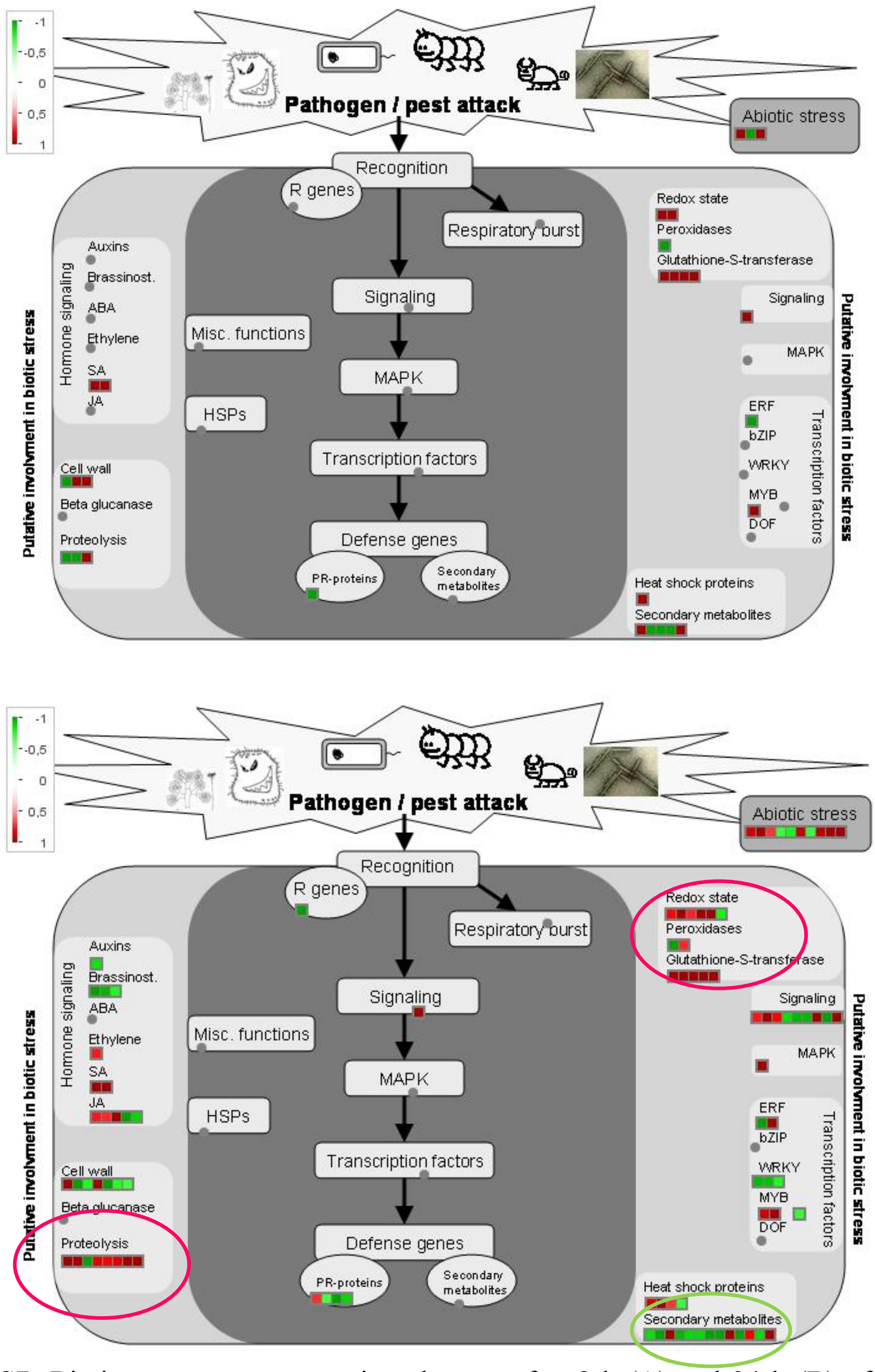


**Figure S7:** Biotic stress gene expression changes after 8 h (**A**) and 24 h (**B**) of incubation with 200 μM PHN, analyzed using the MapMan tool. Circles highlight major stress responses in which genes are induced (red) or repressed (green). Ratios compare PHN-treated samples to control samples.

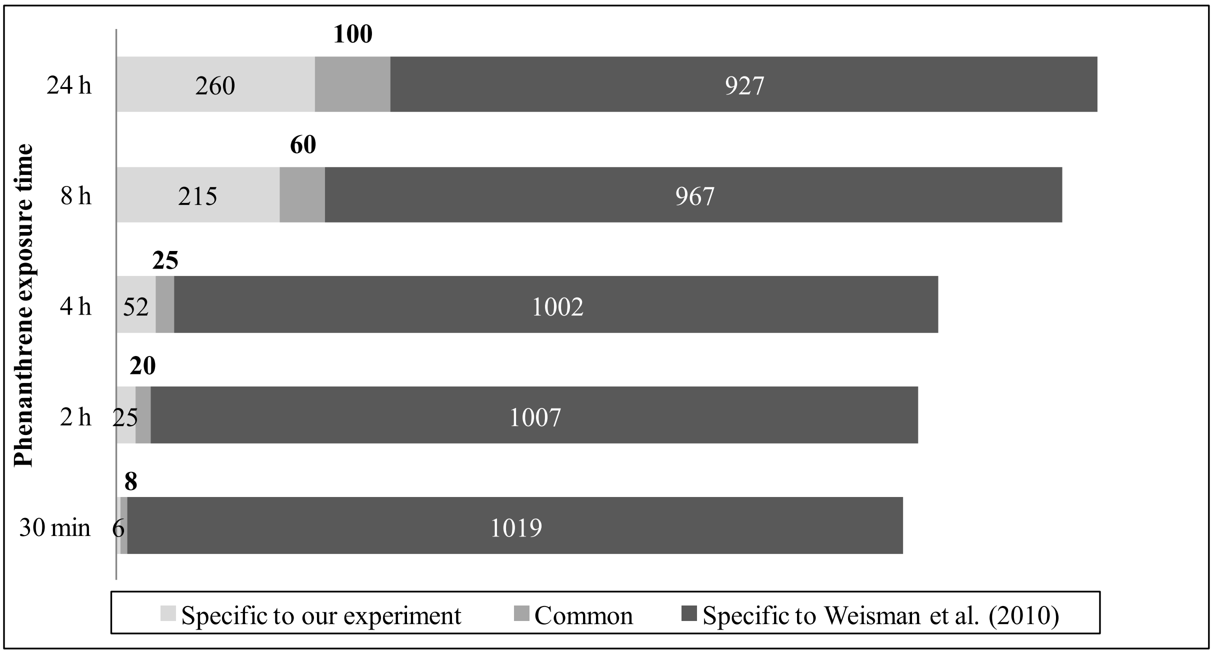


**Figure S8:** Number of differentially expressed genes (DEGs) found specifically in our study or in (Weisman et al., 2010), and shared between both studies. Numbers above histograms indicate the number of DEGs that are shared both experiments at each time point. In all, 14, 45, 77, 275 and 360 genes differentially expressed in our study after 30 min, 2 h, 4 h, 8 h and 24 h of PHN treatment, respectively, were compared to the 1027 DEGs from the Weisman et al. (2010) study.
