# Supplementary material for: Unraveling the early molecular and physiological mechanisms involved in response to phenanthrene exposure
Source: BMC Genomics. 2016 Oct 21;17:818. doi: 10.1186/s12864-016-3133-0 (PMC5073745; doi:10.1186/s12864-016-3133-0)
Supplement: Additional file 3: Figure S9. — Hierachical clustering analysis within Genevestigator public data. (PDF 698 kb) [file 12864_2016_3133_MOESM3_ESM.pdf]

A: Stress selection

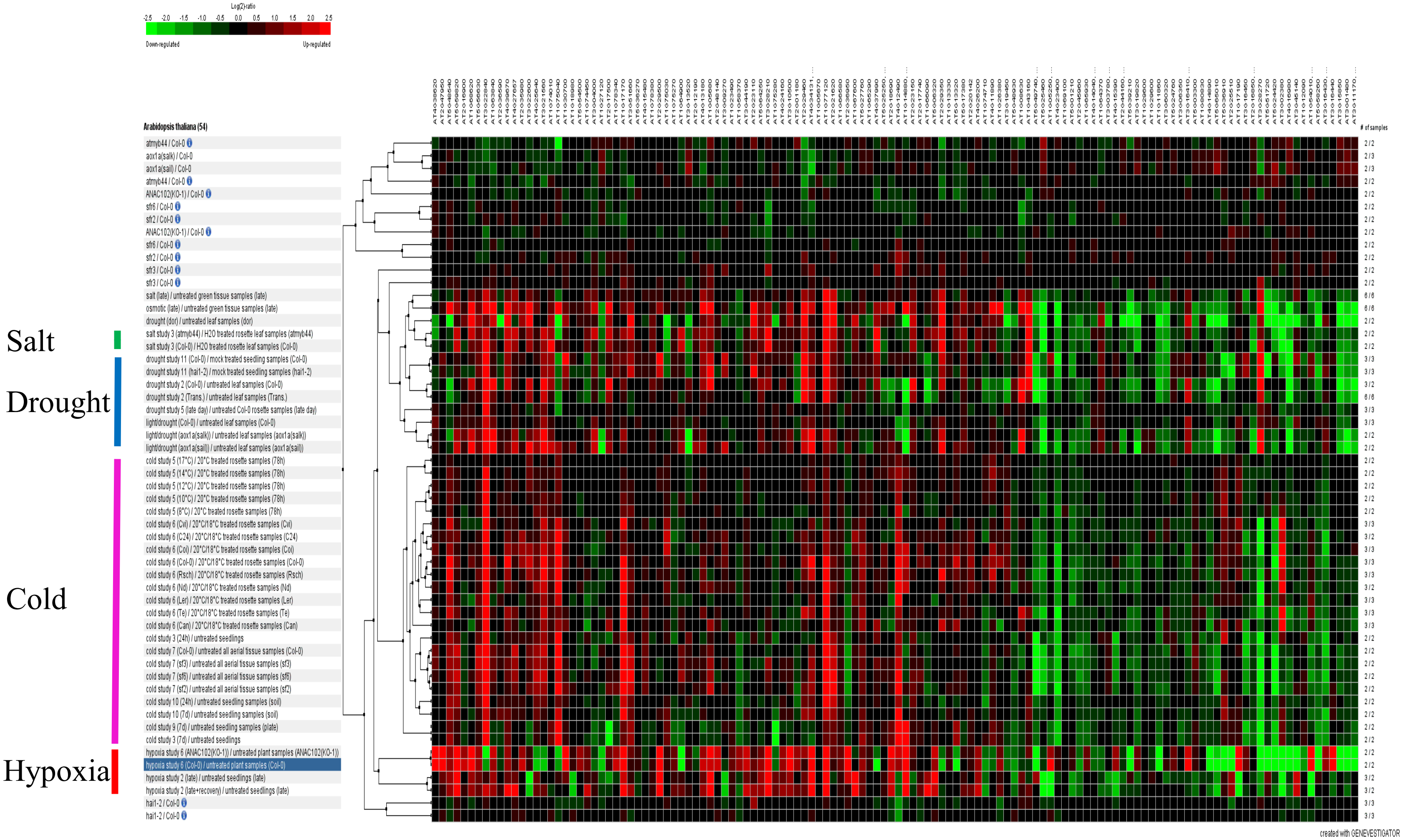

B: biotic selection

Wild type, mutant and transgenic plants response to mutated avirulent or non pathogenic bacteria and fungus

Wild type, mutants and transgenic response to pathogens: virus, bacteria, fungi

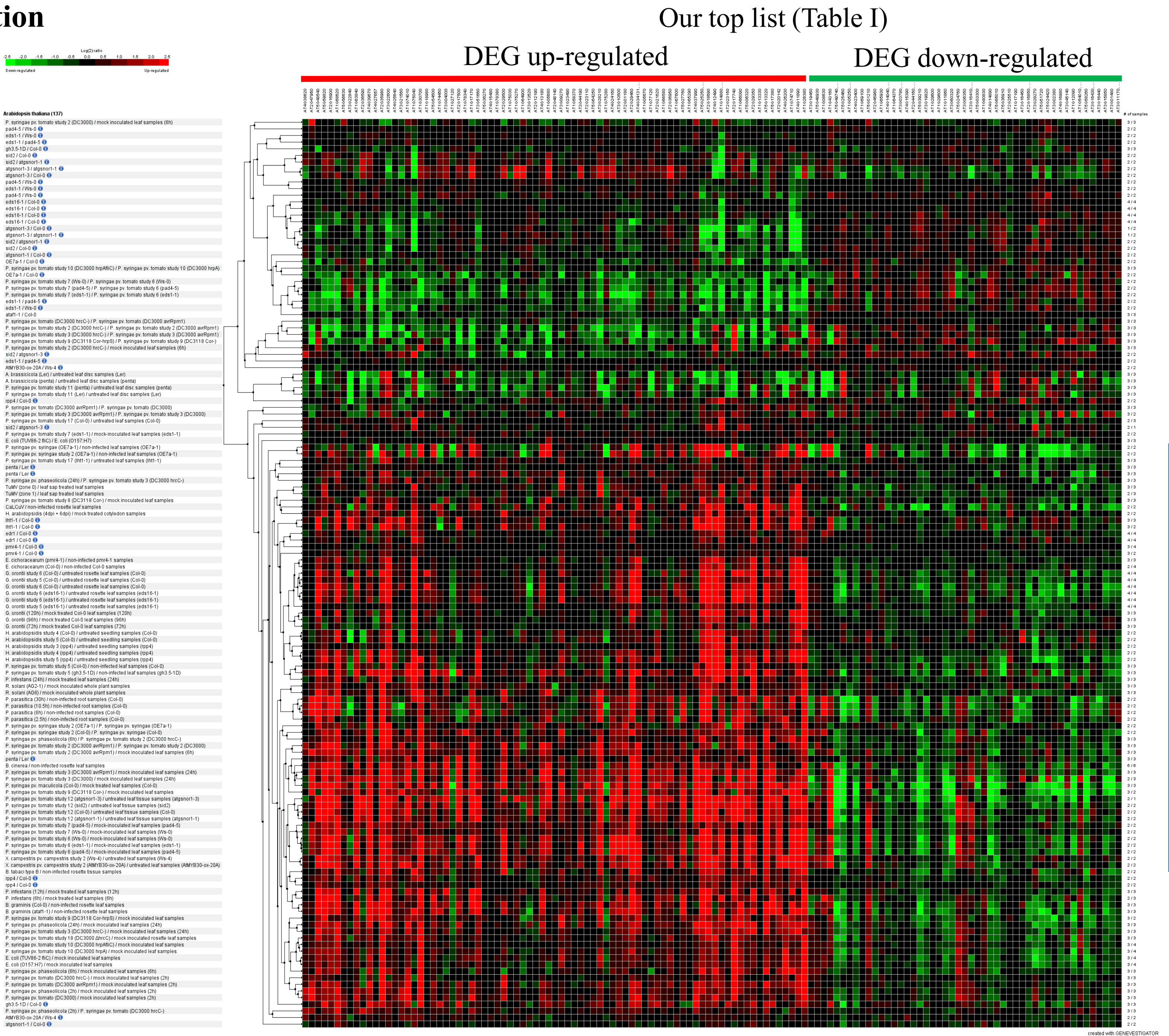

C: Chemical selection

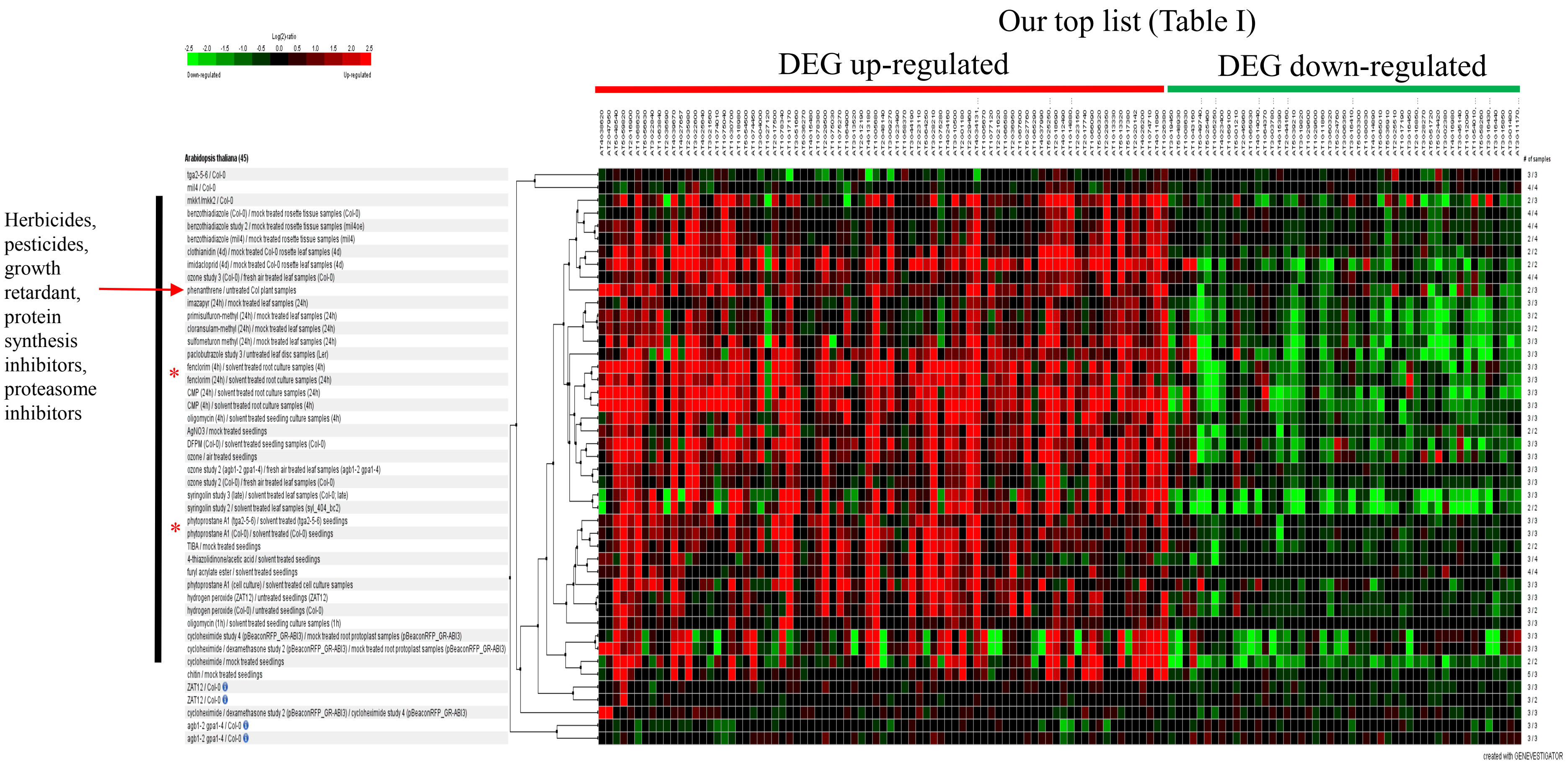

Figure S9: Hierarchical clustering analysis within Genevestigator public data.
Clustering analysis was performed using our top list of differentially regulated genes (Table I) using the clustering tool available at www.genevestigator.com. (**A**) Clustering using subset "Stress selection", (**B**) clustering using subset "Biotic selection", (**C**) clustering uisng subset "chemical stress selection". Red arrow: Weisman et al. (2010) study.*, Denclorim and phytoprostane A1 examples cited in this article.
